# Smart Paper Technology for maternal and newborn health data in The Gambia: A qualitative study of early implementation

**DOI:** 10.1371/journal.pgph.0006244

**Published:** 2026-10-23

**Authors:** Oluwatosin Nkereuwem, Jana Prauseová, Rachel Ford, Alpha Omar Jallow, Musa Marena, Gibril Jarju, Sidat Fofana, Mamady Cham, Buba Manjang, Beate Kampmann, Joanna Busza

**Affiliations:** 1 Vaccines & Immunity Theme, Medical Research Council Unit, The Gambia at the London School of Hygiene and Tropical Medicine, Fajara, The Gambia; 2 Charité Centre for Global Health and Institute for International Health, Charité Universitatsmedizin, Berlin, Germany; 3 London School of Hygiene & Tropical Medicine, London, United Kingdom; 4 Reproductive, Maternal, Newborn Child and Adolescent Health Programme, Ministry of Health, The Gambia; 5 Directorate of Planning and Information, Ministry of Health, Kotu, The Gambia; 6 Expanded Programme on Immunisation, Ministry of Health, Kotu, The Gambia; 7 Bundung Maternal and Child Health Hospital, Kanifing, The Gambia; 8 Directorate of Health Research, Ministry of Health, Kairaba Avenue, The Gambia; ICDDR: International Centre for Diarrhoeal Disease Research Bangladesh, BANGLADESH

## Abstract

Many low- and middle-income countries rely on paper-based maternal and child health records that are often incomplete, fragmented, and difficult to use for continuity of care and decision-making. Smart Paper Technology (SPT), a hybrid paper–digital documentation system, has been proposed as a practical alternative to fully electronic medical records in resource-constrained settings. This study explored the early implementation of SPT in maternal and infant health services in The Gambia, focusing on feasibility and acceptability. We conducted a qualitative descriptive implementation evaluation guided by Rapid Assessment Procedures (RAP) in two pilot facilities in the Western Region of The Gambia between September and December 2024. Data were collected through 30 semi-structured interviews; four focus group discussions with 30 participants spanning facility-level clinical, management, and administrative staff, Ministry of Health staff, and technical and implementing partners; and non-participant observations across key service delivery areas. Data were analysed thematically using NVivo 14. Participants described SPT as feasible and broadly acceptable because it built on familiar paper-based workflows, was introduced through a participatory process, and offered advantages in reporting, information retrieval, and maternal–infant record linkage. However, these benefits were contingent on the implementation context. Key challenges included uneven training coverage, dual documentation, limited internet connectivity, scanning and data capture errors, storage constraints, and concerns about data governance. Staff adapted through peer mentoring, remote troubleshooting, and local workarounds. Participants emphasised that successful scale-up would require sustained training, clear governance arrangements, adequate human and technical resources, and a phased transition to government ownership. SPT offers a practical pathway towards digitisation in maternal and infant health services in LMICs, but its success depends on strong organisational systems, workforce support, and implementation processes that are responsive to routine service realities.

## Introduction

Many low- and middle-income countries (LMICs) face persistent challenges in maternal and child healthcare, particularly during antenatal, delivery, postnatal, and early childhood periods [1–3]. These challenges are often exacerbated by inefficient health information systems, where documentation is incomplete, fragmented across registers, or delayed, undermining continuity of care and timely decision-making [4,5]. The World Health Organization (WHO) emphasises that adequate documentation and robust health information systems are essential for improving service delivery, enabling facilities to track care processes and use routine data for monitoring and quality improvement [3,6]. However, traditional paper-based records remain the norm in many settings and are associated with missing data, transcription errors, weak traceability, and limited access to historical patient information [7,8].

Although digitised medical records offer promising solutions, the transition from paper-based workflows to fully digital platforms is often constrained by high costs, unreliable infrastructure, limited workforce capacity, and sustainability concerns [9–12]. Consequently, hybrid approaches that retain familiar paper-based documentation while enabling structured digital data capture are increasingly adopted in resource-constrained settings [13,14]. Smart Paper Technology (SPT) is one such hybrid solution, using scannable paper forms to support routine data capture and transfer into digital databases for validation, reporting, and use [15,16]. In The Gambia, SPT has been implemented successfully for immunisation services and scaled nationally, demonstrating its operational feasibility within public-sector systems [16–18].

Building on this, the Gambian Ministry of Health piloted SPT within maternal and infant health services at two public facilities [19]. The system allows clinicians to continue using paper at the bedside by replacing handwritten registers with standardised, scannable forms. Once completed, these forms are scanned and processed by an intelligent character recognition (ICR) engine, which converts handwriting into digital data for the national health database.

The rollout followed a participatory design process in which Ministry of Health and facility staff collaborated to ensure the forms mirrored existing clinical workflows. This was followed by intensive onsite training for nurses and midwives. During the transition, the pilot clinics utilised a period of dual record-keeping, maintaining both old registers and new SPT forms, to ensure data safety while the new system was being validated.

However, there is still limited evidence on how SPT performs when introduced into longitudinal maternal and infant health pathways, where documentation occurs across multiple service points, cadres, and time periods. The effectiveness of such tools depends not only on the technology itself, but also on training coverage, alignment with existing workflows, physical infrastructure and internet connectivity, and the responsiveness of implementation support [18]. Together, these factors determine whether hybrid systems close documentation gaps or instead create a dual documentation burden for overstretched staff [13,14]. Understanding early implementation experiences is therefore critical, particularly where national stakeholders are considering scaling SPT across routine maternal and newborn services.

This qualitative study explores the early introduction and adaptation of SPT in two pilot health facilities. We specifically assessed feasibility and acceptability as implementation outcomes. Following Proctor’s framework, we defined feasibility as the extent to which a new treatment, innovation, or evidence-based practice can be successfully implemented within a given context or setting [20]. Acceptability was defined as the perception among implementation stakeholders that a given treatment, service, practice, or innovation is agreeable, palatable, or satisfactory [20]. By focusing on these two dimensions, the study aimed to generate concrete lessons to inform national scale-up, strengthen integration within routine health information systems, and guide policymakers and implementers in The Gambia and similar LMIC settings.

## Materials and methods

### Ethics statement

Ethical approval was obtained from the Gambia Government/MRCG Joint Ethics Committee (Ref: 31096) and the Observational/Interventions Research Ethics Committee of LSHTM (Ref: 31096). All interview and FGD participants received study information and provided written informed consent prior to participation. For non-participant observations, we obtained permission from facility and unit heads. Additionally, researchers verbally informed staff and patients of their presence and the purpose of the observation, ensuring that any individual could decline to be observed without providing a reason.

This study followed the Consolidated Criteria for Reporting Qualitative Research (COREQ) to ensure transparency and rigour in qualitative reporting (S1 File).

### Study design and approach

We conducted a qualitative descriptive implementation evaluation guided by Rapid Assessment Procedures (RAP) to generate timely, practice-relevant evidence on the feasibility and provider acceptability of SPT in routine maternal and infant health services [21,22]. The study was underpinned by a pragmatist research paradigm, which informed the use of multiple methods and prioritised practical, context-relevant findings to support implementation decision-making. RAP was appropriate because the study was embedded within a live pilot and needed to provide rapid, actionable learning for implementers while retaining the depth and transparency expected of qualitative inquiry. Rigour was ensured through multi-method triangulation across interviews, focus groups, and structured observations; purposive sampling to achieve variation across participant groups and implementation roles; iterative team debriefings to verify emerging interpretations and refine data collection guides; and reflexivity maintained throughout fieldwork via reflective field notes and team discussions. This approach is consistent with the consensus-based Planning for and Assessing Rigour in Rapid Qualitative Analysis (PARRQA) framework [23].

### Study setting

The study was conducted in The Gambia’s Western Health Region at two public facilities piloting SPT for maternal and infant health services: Bundung Maternal and Child Health Hospital, a high-volume facility providing maternity and child health care, and Fajikunda Health Centre, a peri-urban facility delivering routine facility-based maternal and child health services. These sites were selected to capture early implementation experiences across contrasting operational contexts and service delivery conditions.

### Intervention description

SPT is a hybrid paper–digital documentation approach in which health workers complete structured, scannable paper forms at the point of care [15,16]. Completed forms are subsequently scanned, and data are extracted into a digital database for verification, storage, and reporting. After scanning, completed SPT forms were retained and physically stored at the facility as part of the pilot documentation workflow. In this pilot, SPT was introduced to support routine maternal and infant health documentation and reporting workflows. This included adapting tools to the service context by redesigning existing national registers into scannable formats that captured specific clinical indicators, such as malaria prophylaxis in pregnancy. Implementation also involved staff orientation and training, the provision of scanning hardware, and the establishment of a support system in which technical teams helped staff resolve errors and verified digital data against the original paper forms to ensure accuracy. Full details of the intervention design, pilot implementation, and maternal and infant health documentation workflow are described in the published protocol paper [19]. A completed TIDieR checklist describing the intervention is provided as a supplementary file (S2 File) [24].

### Participants, sampling and recruitment

We used purposive sampling to capture perspectives across roles involved in, or responsible for, the pilot introduction and routine use of SPT. Eligible participants were adults engaged in maternal and/or infant health documentation, SPT use, supervision, data verification, technical support, or policy and programme oversight relevant to the pilot. Sampling aimed to achieve variation across facility-level clinical staff, facility-level administrative and records staff, facility-level management staff, Ministry of Health staff, and technical and implementing partners to reflect the full implementation pathway from point-of-care documentation to reporting.

Recruitment was coordinated through facility and programme structures. To begin, the research team met with hospital directors and department heads, who provided staff lists and helped identify which clinicians and records officers were actively involved in the SPT pilot. While facility leaders facilitated these initial introductions, the research team then approached these staff members individually to provide study information and invite them to participate. This direct contact allowed the team to explain that participation was voluntary and confidential, independent of their supervisors’ oversight, which helped mitigate potential selection bias. Participation was scheduled to minimise disruption to clinical services, and all participants provided written informed consent.

### Data collection

Data were collected during the pilot implementation period, from 10 September 2024–12 December 2024, using semi-structured interviews, focus group discussions (FGDs), and structured non-participant observations, consistent with RAP’s emphasis on triangulation and iterative learning. Data collection was organised in phases aligned with key implementation milestones, including the initial distribution of scannable forms to antenatal clinics, the transition from traditional paper registers to scanning and digital upload of records, and the point at which staff began electronically linking maternal and infant records. This allowed the team to document early introduction, initial integration into routine workflows, and subsequent adaptation as staff gained experience and implementation support processes matured.

Semi-structured interviews were conducted by trained research assistants with facility-level clinical staff, facility-level administrative and records staff, and facility-level management staff involved in the introduction and routine use of SPT, to elicit their experiences of implementation, with a specific focus on logistical feasibility (such as infrastructure and technical support) and individual acceptability (including perceived value and workload burden). Interviews were used, rather than focus groups alone, for this group because they allowed for in-depth confidential exploration of individual experiences and concerns (particularly around workload and acceptability), which facility-level staff may have been reluctant to share openly in group settings. Interviews were conducted in private settings at or near participants’ workplaces, where feasible, and were scheduled to minimise disruption to service delivery. Each interview lasted between 25 and 41 minutes, with most averaging 35 minutes, allowing sufficient time to cover the full topic guide (S1 Text) while also allowing participants to raise unanticipated issues. All interviews were conducted in English. Interviews were audio-recorded and transcribed verbatim. The topic guide was reviewed and refined during team debriefs to ensure that emerging implementation issues were explored consistently across cadres and stakeholder levels without compromising comparability across participants.

Four trained field assistants carried out structured non-participant observations to document how SPT was integrated into routine practice. Observations focused on workflow sequences and documentation steps from consultation to form completion and preparation for scanning. They also examined staff coordination, interruptions, time pressure, staffing constraints, equipment availability, and emergent workarounds, including parallel use of paper registers. Observations were conducted at two time points during early implementation to capture adaptation over time. Each field assistant rotated through service units (registration, ANC, laboratory, delivery, postnatal care, and infant welfare), spending two consecutive days in each unit. A semi-structured guide (S2 Text) was used to ensure consistency across units and sites. Field notes were recorded during sessions and expanded promptly afterwards to preserve detail on workflow sequences, bottlenecks, and operational constraints.

The FGDs were used to capture shared experiences and to explore how perspectives converged or diverged across cadres involved in documentation, supervision, and implementation support. FGDs complemented interviews by enabling discussion across different groups, making them particularly suited for exploring shared understandings, group norms, and collective perspectives on system-level implementation issues. Participants included Ministry of Health staff, technical and implementing partners, facility-level clinical staff, data and information system staff, and facility-level management staff. The FGDs were mixed-group discussions, and facilitators deliberately encouraged contributions across participant categories to reduce the risk that facility-level perspectives would be overshadowed by senior or technical stakeholders. The discussions were facilitated by the lead researcher (ON, a female social scientist) and by trained members of the research team, using a semi-structured guide (S3 Text) informed by findings from interviews and participant observations. The discussions were designed to encourage participation across different cadres of staff and were held in settings that ensured privacy and minimal interruptions. Each FGD lasted between 1 hour 45 minutes and 2 hours 45 minutes, with an average duration of two hours. The discussions were audio-recorded and transcribed, and facilitators maintained structured notes on group dynamics, points of consensus, disagreement, and issues requiring follow-up during subsequent interviews or observations.

Across all methods, the research team held weekly debriefings to synthesise emerging insights, identify areas requiring clarification, and refine prompts for subsequent data collection. This iterative process supported RAP’s purpose of producing timely, implementation-relevant learning while maintaining methodological transparency and coherence across the dataset.

### Research team and reflexivity

The multidisciplinary research team included social scientists, public health researchers, and trained field assistants with expertise in qualitative research, maternal and newborn health, health systems, and digital health implementation. The lead researcher, ON, is a senior social scientist with a Master of Public Health and extensive experience in qualitative and implementation research and worked closely with the broader team to oversee data collection and analysis. Before the qualitative study began, the team had an established working relationship with the Ministry of Health and was already familiar with the wider SPT initiative. Several team members also had prior experience in maternal and child health research in The Gambia, as well as detailed knowledge of the health system, routine documentation practices, and the sociocultural context, all of which strengthened both data collection and interpretation.

Because SPT was a ministry-led pilot integrated into routine services, we anticipated that some participants, especially facility staff, might feel pressured to provide only “positive” answers or avoid criticism. To mitigate this, we emphasised confidentiality and the voluntary nature of participation, clarified that their involvement would not affect their employment or access to support, and, whenever possible, held interviews and discussions in private spaces.

Reflexive notes were maintained throughout fieldwork, and regular team debriefings were used to consider how researchers’ roles, assumptions, and participants’ expectations might shape questioning, responses, and interpretation, and to adjust prompts to elicit both perceived benefits and implementation challenges.

### Data management and analysis

To prepare the data for analysis, all audio recordings were transcribed verbatim, and any personal information was removed to ensure participant anonymity. Observation notes were also de-identified. All files were stored on password-protected institutional computers accessible only to the research team. In the final report, participant quotes were attributed to broad stakeholder role categories rather than specific job titles or institutions, to protect participant confidentiality while retaining relevant contextual information. To enable readers to verify that quoted material was drawn from a diverse range of participants, unique identifiers have been added to all participant quote attributions in the Results section. Interview quotes are labelled using IDI identifiers (e.g., IDI-01), while FGD quotes are labelled using identifiers that indicate both the FGD and the individual speaker within that group (e.g., FGD1–06, FGD3–04). Observation excerpts are labelled as field notes by researcher source.

We analysed the data thematically using NVivo 14 software, following the approach described by Braun and Clarke [25]. First, the team read through the transcripts and field notes to gain an in-depth understanding of participants’ experiences. We then developed a coding framework based on the study objectives and issues emerging from the data. To enhance analytical rigour, we compared findings across different methods, for example, contrasting interview accounts with observational data, and across different participant groups. We paid close attention to views that differed from the majority to ensure a balanced interpretation. Finally, we grouped codes into broader themes that described how well SPT worked in practice, the challenges staff faced, and what would be needed to support successful national scale-up. The complete coding framework, including codes, sub-themes, themes, and representative exemplar quotes, is provided as a supplementary file (S1 Table).

### Ethics

#### Inclusivity in global research.

Additional information regarding the ethical, cultural, and scientific considerations specific to inclusivity in global research is included in the Supporting Information (S4 Text).

## Results

### Participants and data sources

A total of 30 semi-structured interviews were conducted with facility-level clinical staff, administrative and records staff, and management staff involved in implementation and routine use of SPT in maternal and infant health services (Table 1). In addition, four mixed FGDs were conducted with 30 participants drawn from Ministry of Health staff, technical/implementing partners, facility-level clinical and management staff, and administrative and records staff. The composition of each FGD is shown in Table 2 to provide clearer context on the range of participant roles represented in each discussion. To complement these perspectives, structured observations were carried out across key service delivery areas involved in the pilot. Each participant quote in the Results section carries a unique participant identifier. IDI denotes an in-depth interview. FGD quote identifiers indicate both the FGD and the individual speaker within that group, for example, FGD1–06, FGD3–04, and FGD4–04. Observation excerpts are identified as fieldnotes nu researcher source.

**Table 1:** Composition of participants who were interviewed at the facility level.

Designation	Frequency (N = 30)	Percentage (%)
Facility-level clinical staff	22	73
Facility-level administrative and records staff	5	17
Facility-level management staff	3	10

**Table 2:** Composition of participants who participated in focus-group discussions.

Designation	FGD 1	FGD 2	FGD 3	FGD 4	N (%)
Ministry of Health staff	1	2	4	7	14 (47%)
Technical/implementing partners	3	1	0	0	4 (13%)
Data/information system staff	1	2	1	0	4 (13%)
Facility-level clinical staff	1	3	0	0	4 (13%)
Facility-level management staff	2	0	1	0	3 (10%)
Facility-level administrative staff	0	0	1	0	1 (3%)
Total	8	8	7	7	30 (100%)

### Overview of findings

Fig 1 presents the findings as an RAP-informed implementation pathway, showing how triangulated evidence and iterative feedback informed the interpretation of early implementation experiences and implications for scale-up. The findings are organised under four themes: (i) SPT introduction and adaptation, (ii) implementation challenges, (iii) perceived benefits and drivers of acceptability, and (iv) sustainability and prospects for scale-up. Together, these themes reflect both the practical feasibility of implementation and the factors shaping staff acceptance of SPT in routine care.

**Fig 1:**
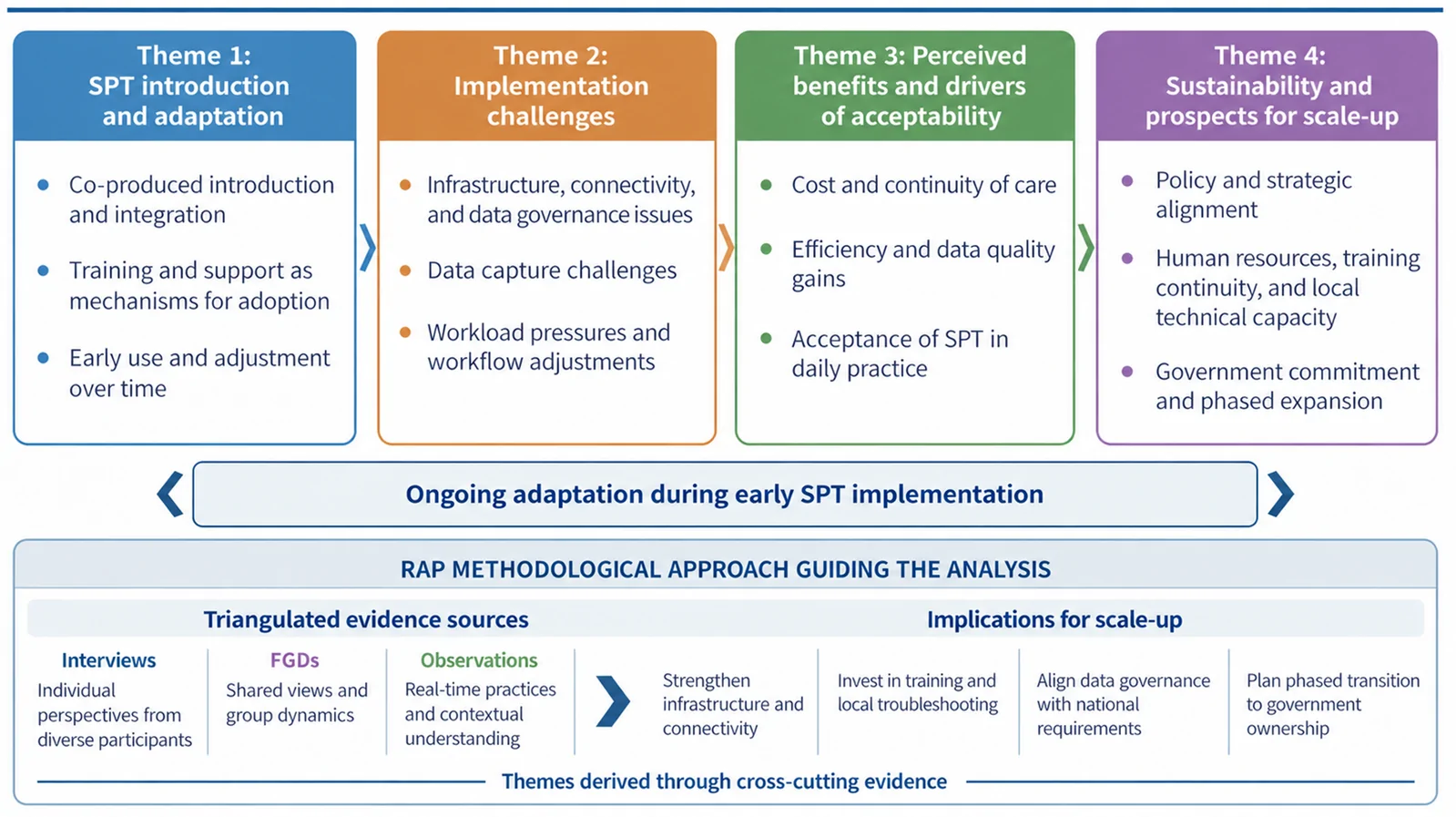
RAP-informed summary of key qualitative findings on early Smart Paper Technology (SPT) implementation in maternal and infant health services in The Gambia. The four themes were identified from triangulated interviews, focus group discussions, and structured observations. The lower panel summarises the Rapid Assessment Procedures (RAP) methodological features underpinning the findings.

1. SPT introduction and adaptation
  1.1 Co-produced introduction and integration

SPT was framed by participants as a co-produced intervention rather than a fixed technology drop-in. They described early engagement as meaningful because it enabled the system to reflect clinical realities and created a basis for legitimacy at the point of use. This sense of contribution was expressed directly:

*"Yes, yes, yes, a lot of contributions came from us (health workers). We were asked to share our opinion... which formed part of the development." (Facility-level management staff, FGD1-06)*

Participants explained that SPT was based on existing documentation tools and improved through feedback. As a result, they viewed it as a familiar system adapted to their routine work, rather than a completely new tool imposed from outside:

*"The materials used were initially taken from the existing tools, the HMIS (Health Management information System) tools, and then, after certain verifications, the health workers were the ones who made contributions on what they would like to work with in their facility." (Technical/implementing partner, FGD1-05)*

However, participants also noted that participatory design did not resolve all existing weaknesses. Rather, it highlighted important omissions in the forms, especially where key maternal procedures were missing. These gaps raised concerns about clinical completeness and required further revision.

*“All other surgeries like hysterectomy, myomectomy, and evacuation were not captured in the SPT. It was a concern I raised with the SPT team, and said they will develop forms for those also, or incorporate those surgeries inside the forms.” (Facility-level management staff, IDI-14)*

Frontline accounts suggested that early mismatches between SPT variables and established registers created uncertainty about which source is correct, particularly during the transition period when staff were still calibrating the new system against longstanding documentation routines:

*"So, there was a push and pull, and some of the variables were not added. Not all the variables that are in the book are on the SPT." (Facility-level management staff, FGD3-04)*

Overall, these accounts suggest that SPT integration was a gradual process in which staff initially had to navigate both existing documentation practices and the new forms. As gaps became apparent, feedback from routine users informed revisions to improve usability and clinical completeness.

1.2 Training and support as mechanisms for adoption

Training functioned as a key mechanism shaping early implementation quality. Where training was structured and practical, staff described it as building procedural confidence through repeated guided practice, not just orientation. One participant highlighted the intensity and learning-by-doing format:

*"Before we started using the technology, we sometimes spent … hours training on SPT. The developer would guide us through various... forms... Everyone [was] assigned to fill in the various forms individually, with a screen display to guide and direct us in the process…" (Facility-level administrative and records staff, IDI-02)*

In contrast, participants at one facility described uneven training coverage due to staff absences, which required informal workarounds:

*“Yes, there was training before the start, but not all of us were trained… Some were on leave, others travelled, so when we started it, we had a little bit of a problem, and I can say we still have some of those problems.” (Facility-level clinical staff, IDI-17)*

These gaps shifted learning responsibility from formal training structures to colleagues and supervisors, resulting in peer-led micro-training that helped sustain implementation but also risked variability in practice across cadres and units:

*“No! There was no organised training from the hospital… But our senior colleagues trained us in their own local ways to make us understand it.” (Facility-level clinical staff, IDI-09)*

Field documentation similarly indicated that cross-site learning and peer support were mobilised deliberately to minimise service disruption rather than waiting for ideal training conditions:

*“The first hospital that adopted the SPT was very involved in supporting and training the second health facility, creating innovative ways to minimise disruption to routine hospital activities.” (Field notes, researcher #2)*

Participants also described remote support structures (e.g., WhatsApp) as a pragmatic extension of training and troubleshooting, particularly where routine clinical workload made in-person sessions difficult to coordinate:

*"Sometimes, we also do online group training. We created an account and a WhatsApp group, where we also receive training occasionally. Because it is normally difficult for everyone to be present at the training ground, we decided to introduce a WhatsApp training group to explain to us." (Facility-level administrative and records staff, IDI-01)*

1.3 Early use and adjustment over time

Early use was characterised by a transition period in which uncertainty about feasibility and sustainability shaped staff responses, particularly where documentation expectations were already high. Accounts suggested that initial difficulty was not simply resistance to change, but a predictable adjustment as staff learned a new precision-based form-filling process:

*“By the time they said SPT is coming, all the staff were afraid.” (Facility-level clinical staff, IDI-05)*

*“The start was very complicated… It was hard for the first month, but as things went on, it became easier." (Facility-level administrative and records staff, IDI-01)*

Concerns also extended to whether SPT would last long enough to justify the effort of adaptation:

*“My initial thought was like… is it actually going to work? Like, is it actually going to continue, or will they withdraw it later?” (Facility-level clinical staff, IDI-27)*

Observations during early implementation helped contextualise these perceptions by showing how documentation competed with patient-facing work under real clinic pressures:

*“Staff frequently struggled to balance patient care with the additional documentation time required for SPT. In some clinics, forms piled up and were completed in batches at the end of the day.” (Field notes, researcher #1)*

Practical usability issues, such as limited writing space and the need to repeat entries after mistakes, increased early frustration and slowed documentation:

*“So, the initial challenge that I encountered was that the boxes were very small. Sometimes you would start to enter up until the last point, and you’ll make a mistake. So, you will have to cancel all that and start again, and that was very tedious.” (Facility-level clinical staff, IDI-10)*

Over time, as users became more familiar with the process, confidence and efficiency improved, and many health workers described the system as easier to use than they had expected once they adapted to it.

2. Implementation challenges
  2.1 Infrastructure, connectivity, and data governance issues

Across both pilot sites, infrastructure constraints shaped whether SPT functioned as a time-saving system or an additional burden. Unreliable internet connectivity was repeatedly described as the most disruptive barrier, undermining timely syncing and creating uncertainty about whether records had been successfully captured:

*"The only problem we have is a network [internet] problem. If you register a person today, before it gets updated, it [could] take [up to] two months sometimes." (Facility-level administrative and records staff, IDI-01)*

*"Yes! The network [internet] is always a problem. Everybody knows the network here... At times, you will have to register the person again, which is not the aim of the project." (Facility-level administrative and records staff, IDI-03)*

When syncing delays occurred, staff described being unable to retrieve information when needed, reinforcing perceptions that system performance was dependent on factors outside frontline control:

*"Some of the challenges are with the records [department]. When you need information or data... they will tell you that the data is not yet synced, or at times they will tell you that there is no internet connection." (Facility-level management staff, IDI-07)*

Participants also noted that storing completed paper forms after scanning could become difficult as record volumes increased, especially where secure storage space was limited.

*“We also need more space, so that any document we scan can be kept safe. There is space, but it is not enough.” (Facility-level administrative and records staff, IDI-02)*

Beyond connectivity and storage space, higher-level stakeholders also raised concerns about data governance. In particular, they emphasised that long-term sustainability would require alignment with national expectations around data hosting, server infrastructure, and data protection.

*“The infrastructure needs to be on the Gambian servers because if not... there will be lots of questions and you will even have to face lots of challenges… because of these data protection laws.” (Ministry of Health staff, FGD4-03)*

2.2 Data capture challenges

Participants also described data capture challenges related to how forms were completed and read by the scanner. Problems such as illegible handwriting, faint ink, or writing outside the required spaces sometimes meant that forms could not be read accurately and had to be corrected or re-entered:

*"With the SPT, you are constrained. There is a margin that you should not go beyond. So, you need to stop there... Some people’s handwriting is not legible enough for the computer to read it. I think that’s a challenge." (Facility-level management staff, FGD3-04)*

*“During the first month of implementation, several staff members were observed rewriting forms that the scanner could not read due to faint ink or slightly misaligned writing. In one clinic, staff kept “correction folders” where they stored forms that needed to be re-entered later.” (Field notes, researcher #2).*

Where variable duplication existed across forms, staff interpreted this as inefficient design that increased effort without clear added value, particularly when the same information was captured in multiple places:

*"One quick challenge … is that there are duplications of variables in certain SPT forms. For example, looking at the antenatal form, you have lab variables there, and in the lab form, you also have the same variables." (Facility-level administrative staff, FGD3-03)*

In addition, some staff reported discrepancies between paper entries and system-generated outputs after scanning, raising concerns about reliability and trust in automated reporting, especially because routine reporting depends on the perceived correctness of aggregated figures:

*"The SPT helps to generate the reports, but the challenge we have is that... there are some inconsistencies when it comes to numbers, on the paper and the report on the system." (Facility-level clinical staff, IDI-27)*

2.3 Workload pressures and workflow adjustments

A major challenge during implementation was the period of dual documentation. Staff described this as increasing their workload, reducing time for patient care, and pushing documentation into after-hours work. Although this was intended as a temporary transition, its continuation reduced acceptance of SPT because staff could not yet experience the system as saving time or improving efficiency:

*“We are doing the SPT and again recording in our registers just like how we were doing it before, with the SPT now adding another problem for us.” (Facility-level clinical staff, IDI-17)*

This burden was particularly salient in high-volume units and during long shifts, where the “end of shift” did not equate to completion of documentation obligations:

*“The reason why I said that it is time-consuming is that after closing from a night shift working for 12 hours tirelessly, from 20:00 to 08:00, and before going home, you must complete these forms depending on the number of deliveries you have conducted.” (Facility-level clinical staff, IDI-07)*

*“In both hospitals, documentation often continued after patient care hours, and staff were seen dividing tasks among themselves, one providing care, another catching up on SPT entries.” (Field notes, researcher #2)*

Overall, the workflow burden was uneven and most acute in high-volume, understaffed units. Under these conditions, completing SPT documentation after clinical care became time-consuming, indicating that successful integration depends on adequate staffing and task-sharing.

3. Perceived benefits and drivers of acceptability
  3.1 Cost and continuity of care

Despite early barriers, participants increasingly evaluated SPT against the costs of paper registers and the strategic aspiration to move toward digitisation. Some framed SPT as a financially realistic reform in a resource-constrained system, describing it as a mechanism to reduce long-term printing and operational costs while improving data integrity:

*“To me, this will have a transformative impact since it is cost-effective, and we all know that the financing when it comes to health is very low… This will minimise a lot of costs in almost all the health facilities in the region, and it will also help in data completeness and data integrity.” (Ministry of Health staff, FGD3-02)*

Drawing on earlier national experience with SPT in immunisation services, one respondent contrasted previous printing costs with the lower costs of running SPT:

*“During the national evaluation for the Expanded Programme on Immunisation component, the annual printing cost was higher. However, with SPT, the cost is now less… So, it is far cheaper to operationalise SPT than the conventional printing cost.” (Technical/implementing partner, FGD1-05)*

Participants described the linkage of maternal and infant records as a practical advantage because it made patients easier to trace, reduced the risk of lost identifiers, and supported follow-up and continuity of care:

*“One of the advantages is that the mother’s and child’s numbers are linked. If a child loses their card and the mother is registered, you can access the mother’s SPT record and find the linked child’s number.” (Facility-level clinical staff, IDI-23)*

*“If you have the correct SPT number, you can see the woman’s antenatal visits, when and where she delivered, and the baby’s information after delivery.” (Facility-level management staff, FGD3-04)*

3.2 Efficiency and data quality gains

Once staff became more familiar with SPT, its efficiency benefits became clearer. Participants highlighted faster retrieval of information, less dependence on paper records that could be lost or damaged, and easier reporting. In contrast, paper registers were described as more fragile and less accessible:

*“Because the SPT is in the system, with just a click, you will get access to whatever you want to check. But the books can go missing, they can tear off, and they can be misplaced”. (Facility-level clinical staff, IDI-15)*

Some participants described speed as both an efficiency outcome and a marker of adaptation, particularly for high-volume documentation tasks:

*“With the SPT, you can record more than 30 prescription pads within an hour, making it easier. It is faster, and it requires less time, compared to the register; it is very different”. (Facility-level administrative and records staff, IDI-28)*

Automated reporting was also described as shifting routine data work away from manual tallying, with implications for timeliness and accountability in service reporting*.*

3.3 Acceptance of SPT in daily practice

Staff acceptance and routine use of SPT grew as confidence increased and support remained available. Participants suggested that hands-on experience, peer support, and troubleshooting helped staff feel more able to use the system in daily practice. Over time, SPT became less of an unfamiliar extra task and more a part of routine work.

Participants described staff support for SPT in relation to the practical advantages it offered in daily work, particularly easier reporting and quicker access to patient information.

***“*** *Before, we used to prepare statistics on a weekly or monthly basis for each condition. SPT has made it easier for us by the end of every month. With SPT, these are automatically generated and segregated. SPT has made life easier for us; that’s the reason why we like using it.” (Facility-level administrative and records staff, IDI-02)*

Our observations also showed that, as staff became more familiar with the system, they used it more confidently. This suggested a shift from early uncertainty to greater ease with SPT.

4. Sustainability and prospects for scale-up
  4.1 Policy and strategic alignment

Many participants described scale-up as both an operational and a governance issue. They emphasised that high-level engagement was needed to move SPT from a pilot project to an institutional commitment, with clear mandates, funding, and system-wide communication:

*“Stakeholder engagement should be done at a very high level and there should be certain critical decisions to be taken or commitments to be made by the Ministry of Health and the government to ensure that this will continue”. (Ministry of Health staff, FGD3-05)*

Participants in the FGDs viewed policy alignment as supportive of SPT implementation. They noted that SPT was consistent with broader government efforts to strengthen digital health systems and improve routine health information management. However, they also emphasised that policy support alone would not be enough without the infrastructure, training, and technical support needed for implementation:

*“Currently, I think the policy of the Ministry is in line with the SPT… policy-wise, I think the policies are in favour of the SPT.” (Ministry of Health staff, FGD3-06)*

4.2 Human resource, training continuity, and local technical capacity

SPT did not remove the need for data work; rather, it redistributed it and made certain roles, such as scanning, verification, synchronisation, and troubleshooting, more important. Shortages in these areas were seen as a direct threat to sustainability:

*“We need to have human resources. The data managers are not there. And the necessary equipment needs to be there.” (Ministry of Health staff, FGD4-02)*

*“[Take] consideration of [an] adequate number of data entry clerks… we need the right resources there.” (Ministry of Health staff, FGD4-04)*

Ongoing capacity-building was also seen as essential for replication and system stability, particularly if SPT is expanded to facilities with less implementation support:

*“There should always be continuous training of workers who can replicate the SPT system.” (Ministry of Health staff, FGD4-01)*

*“Quarterly training… about four to five trainings in a year… [would] actually help in mastering SPT.” (Technical/implementing partner, FGD1-03)*

There was also emphasis on strengthening local problem-solving capacity so that technical issues could be addressed without prolonged dependence on external actors.

4.3 Government commitment and phased expansion

Long-term sustainability was closely linked to domestic financing and a planned transition from donor-supported piloting to government ownership. This was described as requiring early clarity on government commitment and deliberate planning for continuity as external support declines:

*“There should be a stakeholder engagement to know the level of commitment from the Ministry of Health and the government as a whole… [and] preparation for transitioning when the project or the funding donors are about to phase out.” (Ministry of Health staff, FGD3-05)*

Scale-up was also seen as feasible if evidence from the pilot sites was used to guide a phased approach, with attention to sensitisation and the time needed for acceptance to develop:

*“Once we can see a very positive outcome from the two facilities as a pilot, then the government would now have to take it up… government and other partners will put in resources to maintain the system.” (Ministry of Health staff, FGD3-06)*

*“When we want to scale to other hospitals… they need to sensitise first… because acceptance is the key challenge we always have.” (Ministry of Health staff, FGD4-06)*

The participants’ accounts also suggested that peer influence could support implementation during expansion, with confident early users acting as local champions.

## Discussion

This qualitative study explored the early implementation of SPT in maternal and infant health services in two public health facilities in The Gambia. We found that SPT was viewed as feasible and acceptable largely because it was built on familiar documentation practices, introduced through a participatory process, and offered visible advantages in reporting and record retrieval. However, feasibility was contingent on implementation conditions, including the extent and continuity of training, the availability of on-site support, the reliability of connectivity and scanning processes, and the amount of parallel documentation required. Similar implementation dynamics have been reported for hybrid paper-digital systems in other LMIC settings, where the benefits of digitisation can be undermined by workflow duplication and infrastructure constraints [26–28].

SPT’s hybrid design allowed facility-level clinical staff to maintain a familiar paper-based workflow at the point of care while enabling digital data processing afterward. Many participants viewed this as a practical alternative to fully electronic medical records in settings where internet connectivity is unreliable and information and communication technology (ICT) capacity is limited. Because SPT aligned with existing routines, it appeared to support early uptake and reduce resistance to a new documentation system. This is consistent with evidence that digital health interventions are more likely to be adopted when they fit established work practices and minimise additional documentation burdens [29,30]. However, the usefulness of digitised outputs depended on reliable scanning, verification, and timely feedback to support data quality and user trust. Digital readiness is therefore not only a technical issue but also an organisational and workforce one, requiring investment in training, support systems, and ongoing alignment with clinical practice as systems evolve [29,30].

Implementation challenges primarily centred on training coverage, workflow complexity, and infrastructure. While participants found the initial training and mentorship helpful, the lack of regular refresher training and uneven exposure across staff led to variable competence and confidence in using the system. Parallel documentation, particularly when SPT did not fully replace existing registers, was widely seen as increasing workload and creating opportunities for inconsistencies across records. These issues mirror those reported in other digital health rollouts where dual systems persist during transition, and implementation relies heavily on a small number of trained staff [31–33]. Despite these constraints, both facilities adapted through peer mentoring, WhatsApp-based troubleshooting, and local workarounds to sustain operations. This experience highlights how adaptive capacity and local problem-solving were crucial for maintaining momentum [34,35].

Perspectives varied across participant groups in ways that reflected their positions within the implementation pathway. Facility-level clinical staff focused on ease of use, time pressures, and the operational implications of scanning delays and duplicate documentation [36]. In contrast, administrative and management staff emphasised data completeness, accountability, and the potential of SPT to strengthen routine reporting and decision-making. This pattern is consistent with wider digital health literature indicating that end-users tend to prioritise workflow implications, whereas higher-level actors focus more on information outputs and system performance [37]. Despite these differences, there was a broad agreement that scale-up would require strong support structures, including ongoing training, clear governance arrangements, and investments in ICT and health information systems to avoid overburdening staff and compromising data quality [38,39].

Beyond implementation dynamics observed at facility level, participants highlighted systemic constraints that SPT alone cannot resolve, including staffing shortages, inconsistent internet connectivity, and limited ICT infrastructure. Governance concerns around data ownership, access, and long-term stewardship were also raised, particularly where external technical partners or cross-border data processing may be involved [40–42]. These systemic factors require deliberate policy attention alongside investment in the technology itself.

The cost implications of SPT merit consideration. Participants noted that SPT may reduce long-term operational costs compared to conventional paper registers, particularly printing costs, as demonstrated by the national EPI experience [18]. However, upfront implementation costs, including procurement of scanning hardware, staff training, connectivity infrastructure, and ongoing technical support, represent a substantial investment [43,44]. In resource-constrained settings without dedicated implementation funding, these costs could present a barrier to scale-up and sustained operation [39]. Explicit costing studies and economic evaluations will therefore be critical to inform decisions about feasibility and value-for-money at national scale [43].

Several practical lessons emerged. First, staff confidence depended on ongoing, facility-based training and refresher support, particularly where initial training coverage had been uneven. Second, dual documentation may be useful as a temporary transition measure, but it should be clearly time-limited to avoid prolonged workload burden. Finally, implementation is likely to be strengthened by practical supports such as adequate storage, clear standard operating procedures, and defined responsibilities for scanning, verification, and troubleshooting, together with continued user feedback to refine form content and layout.

### Strengths and limitations

As a qualitative study, we did not assess quantitative performance indicators such as documentation completeness, timeliness of data availability, concordance between paper and digital records, or error rates across scanning and verification. These outcomes are being examined in a separate evaluation, which will help determine whether the perceived benefits identified here are matched by measurable improvements in data quality and system performance.

A key strength of this study was the use of the RAP framework to triangulate in-depth interviews, focus groups, and structured observations across frontline, managerial, and policy levels (Fig 1). This multi-source design strengthened the analytical credibility of the findings and allowed us to examine not only perceived acceptability, but also the contextual and operational conditions shaping SPT use in routine maternal and infant care. However, the findings are based on two pilot facilities and may not reflect implementation dynamics in smaller, more rural, or lower-resourced settings where staffing, infrastructure, and supervision arrangements differ.

## Conclusion

This study suggests that SPT is a feasible and broadly acceptable approach to improving routine maternal and infant health documentation in The Gambia. Its acceptability stems from its reliance on familiar paper-based workflows while still enabling downstream digitisation. However, participants stressed that these benefits were contingent on effective implementation, including sustained training and supervision, reliable scanning and connectivity, clear role allocation, and minimised parallel documentation. Overall, the findings indicate that hybrid documentation systems may offer a realistic pathway to digitisation in LMIC settings, provided they are underpinned by strong organisational systems, sufficient workforce support, and robust governance arrangements.

## Supporting information

S1 File COREQ_Checklist: Consolidated criteria for reporting qualitative research (COREQ) Checklist. (PDF)

S2 File TIDieR Checklist: Template for Intervention Description and Replication (TIDieR) Checklist. (PDF)

S1 Table Codebook. (PDF)

S1 Text Interview topic guide. (DOCX)

S2 Text Observation guide. (DOCX)

S3 Text Focus group discussion guide. (DOCX)

S4 Text Inclusivity in global research questionnaire. (PDF)
