# Supplementary material for: Smart Paper Technology for maternal and newborn health data in The Gambia: A qualitative study of early implementation
Source: PLOS Glob Public Health. 2026 Oct 23;6(10):e0006244. doi: 10.1371/journal.pgph.0006244 (PMC13693910; doi:10.1371/journal.pgph.0006244)
Supplement: Supplementary file 2 [file pgph.0006244.s002.pdf]

TIDieR
Template for Intervention Description and Replication

The TIDieR (Template for Intervention Description and Replication) Checklist*:

Information to include when describing an intervention and the location of the information

Item number	Item	Where located **	
		Primary paper (page or appendix number)	Other † (details)
	**BRIEF NAME**		
**1.**	Provide the name or a phrase that describes the intervention.	Pages 3, 5	
	**WHY**		
**2.**	Describe any rationale, theory, or goal of the elements essential to the intervention.	Pages 3-5	
	**WHAT**		
**3.**	Materials: Describe any physical or informational materials used in the intervention, including those provided to participants or used in intervention delivery or in training of intervention providers. Provide information on where the materials can be accessed (e.g. online appendix, URL).	Page 5	
**4.**	Procedures: Describe each of the procedures, activities, and/or processes used in the intervention, including any enabling or support activities.	Pages 5-8	
	**WHO PROVIDED**		
**5.**	For each category of intervention provider (e.g. psychologist, nursing assistant), describe their expertise, background and any specific training given.	Pages 5-8	
	**HOW**		
**6.**	Describe the modes of delivery (e.g. face-to-face or by some other mechanism, such as internet or telephone) of the intervention and whether it was provided individually or in a group.	Pages 5-8	
	**WHERE**		
**7.**	Describe the type(s) of location(s) where the intervention occurred, including any necessary infrastructure or relevant features.	Pages 5-8	

	**WHEN and HOW MUCH**		
**8.**	Describe the number of times the intervention was delivered and over what period of time including the number of sessions, their schedule, and their duration, intensity or dose.	Pages 5-8	
	**TAILORING**		
**9.**	If the intervention was planned to be personalised, titrated or adapted, then describe what, why, when, and how.	Pages 5-8	
	**MODIFICATIONS**		
**10.‡**	If the intervention was modified during the course of the study, describe the changes (what, why, when, and how).	Pagess 5-8	
	**HOW WELL**		
**11.**	Planned: If intervention adherence or fidelity was assessed, describe how and by whom, and if any strategies were used to maintain or improve fidelity, describe them.	Pages 5-8	
**12.‡**	Actual: If intervention adherence or fidelity was assessed, describe the extent to which the intervention was delivered as planned.	N/A	

** **Authors** - use N/A if an item is not applicable for the intervention being described. **Reviewers** – use '?' if information about the element is not reported/not sufficiently reported.

† If the information is not provided in the primary paper, give details of where this information is available. This may include locations such as a published protocol or other published papers (provide citation details) or a website (provide the URL).

‡ If completing the TIDieR checklist for a protocol, these items are not relevant to the protocol and cannot be described until the study is complete.

* We strongly recommend using this checklist in conjunction with the TIDieR guide (see *BMJ* 2014;348:g1687) which contains an explanation and elaboration for each item.

* The focus of TIDieR is on reporting details of the intervention elements (and where relevant, comparison elements) of a study. Other elements and methodological features of studies are covered by other reporting statements and checklists and have not been duplicated as part of the TIDieR checklist. When a **randomised trial** is being reported, the TIDieR checklist should be used in conjunction with the CONSORT statement (see www.consort-statement.org) as an extension of **Item 5 of the CONSORT 2010 Statement.** When a **clinical trial protocol** is being reported, the TIDieR checklist should be used in conjunction with the SPIRIT statement as an extension of **Item 11 of the SPIRIT 2013 Statement** (see www.spirit-statement.org). For alternate study designs, TIDieR can be used in conjunction with the appropriate checklist for that study design (see www.equator-network.org).

Supplementary material: TIDieR-informed intervention description and summary codebook

This supplementary file has been prepared to improve transparency in reporting the Smart Paper Technology (SPT) intervention and the qualitative analysis.

S1 Table. TIDieR-informed description of the SPT intervention

TIDieR element	Description
1. Brief name	Smart Paper Technology (SPT) for maternal and infant health documentation.
2. Why	SPT was introduced to address fragmented, incomplete, delayed, and difficult-to-retrieve paper-based maternal and infant health documentation while offering a pragmatic alternative to fully electronic medical records in settings with infrastructure constraints.
3. What: materials	Standardised scannable paper forms adapted from national maternal and infant health registers; scanning hardware; digital database; intelligent character recognition functionality; verification procedures; storage processes for completed forms; and training and troubleshooting materials.
4. What: procedures	Health workers completed SPT forms at the point of care. Completed forms were scanned by designated records/data personnel, processed using intelligent character recognition, checked against paper forms, and transferred into a digital database for reporting and retrieval.
5. Who provided	Facility clinical staff completed forms; administrative/records staff and data personnel supported scanning and verification; Ministry of Health and implementation partners supported form adaptation, training, supervision, and troubleshooting.
6. How	The intervention was delivered through routine facility workflows, in-person practical training, on-site support, peer mentoring, remote troubleshooting through communication groups, and iterative feedback between facility staff and implementation teams.
7. Where	Bundung Maternal and Child Health Hospital and Fajikunda Health Centre in The Gambia's Western Health Region.
8. When and how much	The pilot was evaluated between September and December 2024. Training and support were provided during early implementation, with additional informal and remote support as issues emerged.
9. Tailoring	Existing national registers were redesigned into scannable forms and adapted through feedback from Ministry of Health and facility staff to better reflect routine service workflows and clinical indicators.
10. Modifications	Early implementation feedback led to identification of missing procedures, duplicated variables, small writing spaces, and data capture issues, which informed further refinement of forms and support processes.
11. Planned fidelity	Fidelity was supported through training, use of structured forms, scanning procedures, verification against original paper forms, supervisory support, and troubleshooting.
12. Actual fidelity	N/A
