# Supplementary material for: Smart Paper Technology for maternal and newborn health data in The Gambia: A qualitative study of early implementation
Source: PLOS Glob Public Health. 2026 Oct 23;6(10):e0006244. doi: 10.1371/journal.pgph.0006244 (PMC13693910; doi:10.1371/journal.pgph.0006244)
Supplement: Supplementary file 3 [file pgph.0006244.s003.pdf]

NVIVO CODE BOOK (SPT Qualitative evaluation)

Main theme	Sub-theme	Files	References	Definition	Example quote
**SPT introduction and adaptation**	Co-produced introduction and integration	30	80	How SPT was introduced, adapted, and integrated into facility systems through consultation, feedback, use of existing HMIS tools, modification of forms, involvement of health workers, and alignment with existing service areas such as antenatal, delivery, OPD, laboratory, EPI, and records.	*"Yes, yes, yes, a lot of contributions came from us (health workers). We were asked to share our opinion... which formed part of the development." (Facility-level management staff)*<br>*"The materials used were initially taken from the existing tools, the HMIS (Health Management information System) tools, and then, after certain verifications, the health workers were the ones who made contributions on what they would like to work with in their facility." (Facility-level administrative and records staff)*<br>*"All other surgeries, like hysterectomy, myomectomy, and evacuation, were not captured in the SPT. It was a concern I raised with the SPT team, and said they will develop forms for those also, or incorporate those surgeries inside the forms." (Facility-level clinical staff) "So, there was a push and pull, and some of the variables were not added. Not all the variables that are in the book are on the SPT." (Facility-level clinical staff)*
	Early use and adjustment over time	32	121	Users' initial experiences with SPT, including confusion, resistance, frustration, fear of mistakes, unfamiliarity with forms, and the gradual process of learning, adaptation, confidence-building, and routine use over time.	*"By the time they said SPT is coming, all the staff were afraid." (Facility-level clinical staff)*<br>*"The start was very complicated… It was hard for the first month, but as things went on, it became easier." (Facility-level administrative and records staff) "My initial thought was like… is it actually going to work? Like, is it actually going to continue, or will they withdraw it later?" (Facility-level clinical staff)*<br>*"Staff frequently struggled to balance patient care with the additional documentation time required for SPT. In some clinics, forms piled up and were completed in batches at the end of the day." (Field notes, researcher #1, antenatal clinic)*<br>*"So, the initial challenge that I encountered was that the boxes were very small. Sometimes you would start to enter up until the last point, and you'll make a mistake. So, you will have to cancel all that and start again, and that was very tedious." (Facility-level clinical staff)*
	Training and support as mechanisms for adoption	34	117	The role of training, supervision, mentoring, refresher sessions, peer support, practical demonstrations, technical guidance, and ongoing support in helping users understand, adopt, and	*"Before we started using the technology, we sometimes spent … hours training on SPT. The developer would guide us through various ... forms... Everyone [was] assigned to fill in the various forms individually, with a*

Main theme	Sub-theme	Files	References	Definition	Example quote
				correctly use SPT in routine practice.	*screen display to guide and direct us in the process…" (Facility-level administrative and records staff)*<br>*"Yes, there was training before the start, but not all of us were trained… Some were on leave, others travelled, so when we started it, we had a little bit of a problem, and I can say we still have some of those problems." (Facility-level clinical staff)*<br>*"No! There was no organised training from the hospital… But our senior colleagues trained us in their own local ways to make us understand it." (Facility-level clinical staff)*<br>*"The first hospital that adopted the SPT was very involved in supporting and training the second health facility, creating innovative ways to minimise disruption to routine hospital activities." (Field notes, researcher #2)*<br>*"Sometimes, we also do online group training. We created an account and a WhatsApp group, where we also receive training occasionally. Because it is normally difficult for everyone to be present at the training ground, we decided to introduce a WhatsApp training group to explain to us." (Facility-level administrative and records staff)*
**Implementation challenges**	Data capture challenges	31	154	Challenges related to the accurate and complete capture of data using SPT, including missing variables, small writing boxes, handwriting recognition issues, incorrect disease or treatment codes, duplicated entries, incomplete forms, and difficulties capturing complex clinical or programme indicators.	*"With the SPT, you are constrained. There is a margin that you should not go beyond. So, you need to stop there... Some people's handwriting is not legible enough for the computer to read it. I think that's a challenge." (Facility-level clinical staff)*<br>*"During the first month of implementation, several staff members were observed rewriting forms that the scanner could not read due to faint ink or slightly misaligned writing. In one clinic, staff kept "correction folders" where they stored forms that needed to be re-entered later." (Field notes, researcher #2, antenatal clinic).*<br>*"One quick challenge … is that there are duplications of variables in certain SPT forms. For example, looking at the antenatal form, you have lab variables there, and in the lab form, you also have the same variables." (Facility-level clinical staff)*<br>*"The SPT helps to generate the reports, but the challenge we have is that... there are some inconsistencies when it comes to numbers, on the paper and the report on the system." (Facility-level clinical staff)*
	Infrastructure, connectivity, and data	23	78	Challenges related to the technical and governance environment required for SPT	*"The only problem we have is a network [internet] problem. If you register a person today, before it gets updated, it*

Main theme	Sub-theme	Files	References	Definition	Example quote
	governance issues			implementation, including internet connectivity, synchronisation delays, scanning and upload processes, integration with DHIS2/HMIS, data security, data ownership, access permissions, server/data storage arrangements, and discrepancies between systems.	*[could] take [up to] two months sometimes." (Facility-level administrative and records staff)* *"Yes! The network [internet] is always a problem. Everybody knows the network here... At times, you will have to register the person again, which is not the aim of the project." (Facility-level administrative and records staff)* *"Some of the challenges are with the records [department]. When you need information or data... they will tell you that the data is not yet synced, or at times they will tell you that there is no internet connection." (Facility-level clinical staff)* *"We also need more space, so that any document we scan can be kept safe. There is space, but it is not enough." (Facility-level administrative and records staff)* *"The infrastructure needs to be on the Gambian servers because if not... there will be lots of questions and you will even have to face lots of challenges… because of these data protection laws." (Ministry of Health officials)*
	Workload pressures and workflow adjustments	33	141	How SPT affected daily work routines, including dual recording with registers, increased documentation burden, patient waiting time, staff shortages, task-shifting, use of students or record clerks, delays in completing forms, and the need to reorganise workflow around SPT processes.	*“We are doing the SPT and again recording in our registers just like how we were doing it before, with the SPT now adding another problem for us.” (Facility-level clinical staff)* *“The reason why I said that it is time-consuming is that after closing from a night shift working for 12 hours tirelessly, from 20:00 to 08:00, and before going home, you must complete these forms depending on the number of deliveries you have conducted.” (Facility-level clinical staff)* *“In both hospitals, documentation often continued after patient care hours, and staff were seen dividing tasks among themselves, one providing care, another catching up on SPT entries.” (Field notes, researcher #1, labour ward)*
**Perceived benefits and drivers of acceptability**	Acceptance of SPT in daily practice	27	69	Positive perceptions, willingness to use, and gradual acceptance of SPT among health workers and stakeholders, including views that the tool is useful, becomes easier over time, is beneficial for routine practice, and is preferable to paper-based systems once users become familiar with it.	*“Before, we used to prepare statistics on a weekly or monthly basis for each condition. SPT has made it easier for us by the end of every month. With SPT, these are automatically generated and segregated. SPT has made life easier for us; that's the reason why we like using it.” (Facility-level administrative and records staff)* *“If you have the correct SPT number, you can see the woman’s antenatal visits, when and where she delivered, and the baby’s information after delivery.” (Facility-level clinical staff)*
	Cost and continuity of	25	80	Perceived benefits of SPT in reducing	*“To me, this will have a transformative impact since it is*

Main theme	Sub-theme	Files	References	Definition	Example quote
	care			paper/register burden, improving record storage, supporting patient tracing, linking maternal and child records, maintaining patient history across visits or facilities, and improving continuity of care through unique patient identifiers.	*cost-effective, and we all know that the financing when it comes to health is very low… This will minimise a lot of costs in almost all the health facilities in the region, and it will also help in data completeness and data integrity." (Facility-level management staff)*<br>*"During the national evaluation for the Expanded Programme on Immunisation component, the annual printing cost was higher. However, with SPT, the cost is now less… So, it is far cheaper to operationalise SPT than the conventional printing cost." (Ministry of Health officials)*<br>*"One of the advantages is that the mother's and child's numbers are linked. If a child loses their card and the mother is registered, you can access the mother's SPT record and find the linked child's number." (Facility-level clinical staff)*
	Efficiency and data quality gains	33	169	Perceived improvements in the speed, accuracy, completeness, accessibility, and usability of health data, including faster retrieval of patient information, automated or easier report generation, reduced manual counting, improved data verification, and better availability of data for decision-making.	*"Because the SPT is in the system, with just a click, you will get access to whatever you want to check. But the books can go missing, they can tear off, and they can be misplaced". (Facility-level clinical staff)*<br>*"With the SPT, you can record more than 30 prescription pads within an hour, making it easier. It is faster, and it requires less time, compared to the register; it is very different". (Facility-level administrative and records staff)*
**Sustainability and prospects for scale-up**	Government commitment and phased expansion	20	47	Issues related to long-term continuation and scale-up of SPT, including government financing, transition from donor or project support, national rollout, phased expansion to other facilities or regions, procurement of equipment and forms, and sustained investment after the pilot phase.	*"There should be a stakeholder engagement to know the level of commitment from the Ministry of Health and the government as a whole… [and] preparation for transitioning when the project or the funding donors are about to phase out." (Facility-level clinical staff),*<br>*"Once we can see a very positive outcome from the two facilities as a pilot, then the government would now have to take it up… government and other partners will put in resources to maintain the system." (Ministry of Health officials)*<br>*"When we want to scale to other hospitals… they need to sensitise first… because acceptance is the key challenge we always have." (Ministry of Health officials)*
	Human resources, training continuity, and local technical capacity	28	97	The human and technical capacity required to sustain SPT, including adequate staffing, continuous training, regional and facility-level technical support, data clerks or trained focal persons, local ICT/HMIS capacity, equipment	*"We need to have human resources. The data managers are not there. And the necessary equipment needs to be there." (Ministry of Health officials)*<br>*"[Take] consideration of [an] adequate number of data entry clerks… we need the right resources there." (Ministry*

Main theme	Sub-theme	Files	References	Definition	Example quote
				maintenance, and reduced dependence on external technical partners.	*of Health officials)*<br>*“There should always be continuous training of workers who can replicate the SPT system.” (Data/information system staff)*<br>*“Quarterly training… about four to five trainings in a year… [would] actually help in mastering SPT.” (Facility-level clinical staff)*
	Policy and strategic alignment	15	43	The extent to which SPT aligns with national health information priorities, digital health strategies, HMIS/DHIS2 systems, Ministry of Health policies, programme reporting requirements, governance structures, and the need for formal policy guidance to support integration and scale-up.	*“Currently, I think the policy of the Ministry is in line with the SPT… policy-wise, I think the policies are in favour of the SPT.” (Ministry of Health officials), “Stakeholder engagement should be done at a very high level and there should be certain critical decisions to be taken or commitments to be made by the Ministry of Health and the government to ensure that this will continue”. (Facility-level clinical staff)*
