# Supplementary material for: Smart Paper Technology for maternal and newborn health data in The Gambia: A qualitative study of early implementation
Source: PLOS Glob Public Health. 2026 Oct 23;6(10):e0006244. doi: 10.1371/journal.pgph.0006244 (PMC13693910; doi:10.1371/journal.pgph.0006244)
Supplement: Supplementary file 5 [file pgph.0006244.s008.pdf]

PLOS Global Public Health

# Smart Paper Technology for maternal and newborn health data in The Gambia: a qualitative study of early implementation

--Manuscript Draft--

| | |
|---|---|
| Manuscript Number: | PGPH-D-26-01044 |
| Article Type: | Research Article |
| Full Title: | Smart Paper Technology for maternal and newborn health data in The Gambia: a qualitative study of early implementation |
| Short Title: | Early SPT implementation in The Gambia |
| Corresponding Author: | Oluwatosin Oizamsi Olumide Nkereuwem, MPH<br>MRC Unit The Gambia at LSHTM: Medical Research Council Unit The Gambia at the London School of Hygiene and Tropical Medicine<br>Fajara, The Gambia GAMBIA |
| Order of Authors: | Oluwatosin Nkereuwem |
| | Jana Prauseová |
| | Rachel Ford |
| | Alpha Omar Jallow |
| | Musa Marena |
| | Gibril Jarju |
| | Sidat Fofana |
| | Mamady Cham |
| | Buba Manjang |
| | Beate Kampmann |
| | Joanna Busza |
| Keywords: | Smart Paper Technology; maternal and newborn health; Digital health; health information systems; The Gambia |
| Abstract: | Many low- and middle-income countries rely on paper-based maternal and child health records that are often incomplete, fragmented, and difficult to use for continuity of care and decision-making. Smart Paper Technology (SPT), a hybrid paper–digital documentation system, has been proposed as a practical alternative to fully electronic medical records in resource-constrained settings. This study explored the early implementation of SPT in maternal and infant health services in The Gambia, focusing on feasibility and acceptability. We conducted a qualitative evaluation guided by Rapid Assessment Procedures in two pilot facilities in the Western Region of The Gambia between September and December 2024. Data were collected through 30 semi-structured interviews; four focus group discussions with 30 participants from the Ministry of Health and collaborating institutions; and non-participant observations across key service delivery areas. Data were analysed thematically using NVivo 14. Participants described SPT as feasible and broadly acceptable because it built on familiar paper-based workflows, was introduced through a participatory process, and offered advantages in reporting, information retrieval, and maternal–infant record linkage. However, these benefits were contingent on the implementation context. Key challenges included uneven training coverage, dual documentation, limited internet connectivity, scanning and data capture errors, storage constraints, and concerns about data governance. Staff adapted through peer mentoring, remote troubleshooting, and local workarounds. Participants emphasised that successful scale-up would require sustained training, clear governance arrangements, adequate human and technical resources, and a phased transition to government ownership. SPT offers a practical pathway towards digitisation in maternal and infant health services in LMICs, but its success depends on strong organisational systems, workforce support, and implementation processes that are responsive to routine service realities. |

Powered by Editorial Manager® and ProduXion Manager® from Aries Systems Corporation

| Additional Information: | |
|---|---|
| **Question** | **Response** |
| **Financial Disclosure**<br><br>Enter a financial disclosure statement that describes the sources of funding for the work included in this submission and the role the funder(s) played. This includes grants and any commercial funding of the work or authors.<br><br>This statement will be typeset if the manuscript is accepted for publication.<br><br>*Please review the submission guidelines and the instructions link below for detailed requirements and guidance.* | This work was supported by the Gates Foundation [INV-048710]. Additional support was provided by the IMmunising PRegnant women and INfants network (IMPRINT) which was co-funded by the MRC and BBSRC and from the Medical Research Council [grant number MR/Y033752/1].<br>The funders had no role in study design, data collection and analysis, decision to publish, or preparation of the manuscript |
| **Competing Interests**<br><br>On behalf of all authors, disclose any competing interests that could be perceived to bias this work.<br><br>This statement will be typeset if the manuscript is accepted for publication.<br><br>*Please review the instructions link below and PLOS Global Public Health's competing interests policy to determine what information must be disclosed at submission.* | MM, GJ, SF, and BM are employees of The Gambia Ministry of Health. MC is an employee of Bundung Maternal and Child Health Hospital. The authors declare that these affiliations did not influence the study design, data collection, analysis, interpretation, or the decision to submit the manuscript for publication. |
| **Data Availability**<br><br>Before publication, Authors are required to make fully available and without restriction all data underlying their findings. Please see our PLOS Data Policy page for detailed information on this policy.<br><br>A **Data Availability Statement**, detailing | The qualitative data underlying the findings of this study are not publicly available because they contain potentially identifiable and sensitive participant information. Public sharing would be inconsistent with the ethical approvals governing the study, including approval from the Gambia Government/MRCG Joint Ethics Committee. De-identified data may be made available from the corresponding author upon reasonable request, subject to review and approval in accordance with participant confidentiality, ethical requirements, and institutional data-sharing procedures. Access may require completion of a data-sharing agreement. |

Powered by Editorial Manager® and ProduXion Manager® from Aries Systems Corporation

| | |
|---|---|
| where the data can be accessed, is required at first submission. Insert your Data Availability Statement in the box below.<br><br>Please see the data reporting section of our submission guidelines for instructions on what you need to include in your Data Availability Statement.<br><br>This statement will be typeset if the manuscript is accepted for publication.<br><br>*PLOS allows rare exemptions to address legal and ethical concerns. If you have legal or ethical restrictions, please use the box below to detail these in full sentences for the Journal team to consider.* | |

Manuscript
Click here to access/download;Manuscript;Manuscript.pdf

# Smart Paper Technology for maternal and newborn health data in The Gambia: a qualitative study of early implementation

Oluwatosin Nkereuwem[1], Jana Prauseová[2], Rachel Ford[3], Alpha Omar Jallow[1], Musa Marena[4], Gibril Jarju[5], Sidat Fofana[6], Mamady Cham[7], Buba Manjang [8], Beate Kampmann[1,2#], Joanna Busza[3#],

[1] Vaccines & Immunity Theme, Medical Research Council Unit, The Gambia at the London School of Hygiene and Tropical Medicine, Fajara, The Gambia

[2] Charité Centre for Global Health and Institute for International Health, Charité Universitatsmedizin, Berlin, Germany

[3] London School of Hygiene & Tropical Medicine, Keppel Street, London, United Kingdom

[4] Reproductive, Maternal, Newborn Child and Adolescent Health Programme, Ministry of Health, The Gambia

[5] Directorate of Planning and Information, Ministry of Health, Kotu, Gambia

[6] Expanded Programme on Immunisation, Ministry of Health, Kotu, The Gambia

[7] Bundung Maternal and Child Health Hospital, Kanifing, The Gambia

[8] Directorate of Health Research, Ministry of Health, Kairaba Avenue, Gambia

[#] Joint senior authors

**Corresponding author:** Oluwatosin Nkereuwem, Vaccines and Immunity Theme, Medical Research Council Unit, The Gambia at the London School of Hygiene and Tropical Medicine, Fajara, The Gambia. Email: oluwatosin.nkereuwem@lshtm.ac.uk.

## Abstract

Many low- and middle-income countries rely on paper-based maternal and child health records that are often incomplete, fragmented, and difficult to use for continuity of care and decision-making. Smart Paper Technology (SPT), a hybrid paper–digital documentation system, has been proposed as a practical alternative to fully electronic medical records in resource-constrained settings. This study explored the early implementation of SPT in maternal and infant health services in The Gambia, focusing on feasibility and acceptability. We conducted a qualitative evaluation guided by Rapid Assessment Procedures in two pilot facilities in the Western Region of The Gambia between September and December 2024. Data were collected through 30 semi-structured interviews; four focus group discussions with 30 participants from the Ministry of Health and collaborating institutions; and non-participant observations across key service delivery areas. Data were analysed thematically using NVivo 14. Participants described SPT as feasible and broadly acceptable because it built on familiar paper-based workflows, was introduced through a participatory process, and offered advantages in reporting, information retrieval, and maternal–infant record linkage. However, these benefits were contingent on the implementation context. Key challenges included uneven training coverage, dual documentation, limited internet connectivity, scanning and data capture errors, storage constraints, and concerns about data governance. Staff adapted through peer mentoring, remote troubleshooting, and local workarounds. Participants emphasised that successful scale-up would require sustained training, clear governance arrangements, adequate human and technical resources, and a phased transition to government ownership. SPT offers a practical pathway towards digitisation in maternal and infant health services in LMICs, but its success depends on strong organisational systems, workforce support, and implementation processes that are responsive to routine service realities.

## Introduction

Many low- and middle-income countries (LMICs) face persistent challenges in maternal and child healthcare, particularly during antenatal, delivery, postnatal, and early childhood periods (1-3). These challenges are often exacerbated by inefficient health information systems, where documentation is incomplete, fragmented across registers, or delayed,

undermining continuity of care and timely decision-making (4, 5). The World Health Organization (WHO) emphasises that adequate documentation and robust health information systems are essential for improving service delivery, enabling facilities to track care processes and use routine data for monitoring and quality improvement (3, 6). However, traditional paper-based records remain the norm in many settings and are associated with missing data, transcription errors, weak traceability, and limited access to historical patient information (7, 8).

Although digitised medical records offer promising solutions, the transition from paper-based workflows to fully digital platforms is often constrained by high costs, unreliable infrastructure, limited workforce capacity, and sustainability concerns (9-12). Consequently, hybrid approaches that retain familiar paper-based documentation while enabling structured digital data capture are increasingly adopted in resource-constrained settings (13, 14). Smart Paper Technology (SPT) is one such hybrid solution, using scannable paper forms to support routine data capture and transfer into digital databases for validation, reporting, and use (15, 16). In The Gambia, SPT has been implemented successfully for immunisation services and scaled nationally, demonstrating its operational feasibility within public-sector systems (16-18).

Building on this, the Gambian Ministry of Health (MoH) piloted SPT within maternal and infant health services at two public facilities (19). The system allows clinicians to continue using paper at the bedside by replacing handwritten registers with standardised, scannable forms. Once completed, these forms are scanned and processed by an intelligent character recognition (ICR) engine, which converts handwriting into digital data for the national health database.

The rollout followed a participatory design process in which MoH and facility staff collaborated to ensure the forms mirrored existing clinical workflows. This was followed by intensive onsite training for nurses and midwives. During the transition, the pilot clinics utilised a period of dual record-keeping, maintaining both old registers and new SPT forms, to ensure data safety while the new system was being validated.

However, there is still limited evidence on how SPT performs when introduced into longitudinal maternal and infant health pathways, where documentation occurs across multiple service points, cadres, and time periods. The effectiveness of such tools depends not only on the technology itself, but also on training coverage, alignment with existing

workflows, physical infrastructure and internet connectivity, and the responsiveness of implementation support (18). Together, these factors determine whether hybrid systems close documentation gaps or instead create a "dual-documentation" burden for overstretched staff (13, 14). Understanding early implementation experiences is therefore critical, particularly where national stakeholders are considering scaling SPT across routine maternal and newborn services.

This qualitative study explores the early introduction and adaptation of SPT in these two pilot health facilities. We specifically assessed feasibility, defined as the practical and logistical capability of the health system to support the intervention, and acceptability, referring to the attitudes and willingness of healthcare providers to adopt the tool into their routine work. By focusing on these two dimensions, the study aimed to generate concrete lessons to inform national scale-up, strengthen integration within routine health information systems, and guide policymakers and implementers in The Gambia and similar LMIC settings.

## Materials and methods

### Study design and approach

We conducted a qualitative evaluation guided by Rapid Assessment Procedures (RAP) to generate timely, practice-relevant evidence on the feasibility and provider acceptability of SPT in routine maternal and infant health services (20, 21). RAP informed a pragmatic, implementation-focused design using multi-method triangulation, team-based fieldwork with regular debriefings, and iterative sense-making. This enabled continuous refinement of lines of inquiry as implementation progressed, while preserving sufficient analytic depth for robust thematic interpretation.

### Study setting

The study was conducted in The Gambia's Western Health Region at two public facilities piloting SPT for maternal and infant health services: Bundung Maternal and Child Health Hospital, a high-volume facility providing maternity and child health care, and Fajikunda Health Centre, a peri-urban facility delivering routine facility-based maternal and child health services. These sites were selected to capture early implementation experiences across contrasting operational contexts and service delivery conditions.

## Intervention description

SPT is a hybrid paper–digital documentation approach in which health workers complete structured, scannable paper forms at the point of care (15, 16). Completed forms are subsequently scanned, and data are extracted into a digital database for verification, storage, and reporting. After scanning, completed SPT forms were retained and physically stored at the facility as part of the pilot documentation workflow. In this pilot, SPT was introduced to support routine maternal and infant health documentation and reporting workflows. This included adapting tools to the service context by redesigning existing national registers into scannable formats that captured specific clinical indicators, such as malaria prophylaxis in pregnancy. Implementation also involved staff orientation and training, the provision of scanning hardware, and the establishment of a support system in which technical teams helped staff resolve errors and verified digital data against the original paper forms to ensure accuracy (19).

## Participants, sampling and recruitment

We used purposive sampling to capture perspectives across roles involved in, or responsible for, the pilot introduction and routine use of SPT. Eligible participants were adults engaged in maternal and/or infant health documentation, SPT use, supervision, data verification, technical support, or policy and programme oversight relevant to the pilot. Sampling aimed to achieve variation across frontline providers, facility and records administration, Ministry of Health stakeholders, and IT/data support personnel to reflect the full implementation pathway from point-of-care documentation to reporting.

Recruitment was coordinated through facility and programme structures. To begin, the research team met with hospital directors and department heads, who provided staff lists and helped identify which clinicians and records officers were actively involved in the SPT pilot. While facility leaders facilitated these initial introductions, the research team then approached these staff members individually to provide study information and invite them to participate. This direct contact allowed the team to explain that participation was voluntary and confidential, independent of their supervisors' oversight, which helped mitigate potential selection bias. Participation was scheduled to minimise disruption to clinical services, and all participants provided written informed consent.

## Data collection

Data were collected during the pilot implementation period, from 10 September 2024 to 12 December 2024, using semi-structured interviews, focus group discussions (FGDs), and structured non-participant observations, consistent with RAP's emphasis on triangulation and iterative learning. Data collection was organised in phases aligned with key implementation milestones, including the initial distribution of scannable forms to antenatal clinics, the transition from traditional paper registers to scanning and digital upload of records, and the point at which staff began electronically linking maternal and infant records. This allowed the team to document early introduction, initial integration into routine workflows, and subsequent adaptation as staff gained experience and implementation support processes matured.

Semi-structured interviews were conducted by trained research assistants with facility-level staff involved in the introduction and routine use of SPT, to elicit their experiences of implementation, with a specific focus on logistical feasibility (such as infrastructure and technical support) and individual acceptability (including perceived value and workload burden). Interviews were conducted in private settings at or near participants' workplaces, where feasible, and were scheduled to minimise disruption to service delivery. Each interview lasted between 25 and 41 minutes, with most averaging 35 minutes, allowing sufficient time to cover the full topic guide (S1 Text) while also allowing participants to raise unanticipated issues. All interviews were conducted in English. Interviews were audio-recorded and transcribed verbatim. The topic guide was reviewed and refined during team debriefs to ensure that emerging implementation issues were explored consistently across cadres and stakeholder levels without compromising comparability across participants.

Four trained field assistants carried out structured non-participant observations to document how SPT was integrated into routine practice. Observations focused on workflow sequences and documentation steps from consultation to form completion and preparation for scanning. They also examined staff coordination, interruptions, time pressure, staffing constraints, equipment availability, and emergent workarounds, including parallel use of paper registers. Observations were conducted at two time points during early implementation to capture adaptation over time. Each field assistant rotated through service units (registration, ANC, laboratory, delivery, postnatal care, and infant welfare), spending two consecutive days in each unit. A semi-structured guide (S2 Text) was used to ensure consistency across units and

sites. Field notes were recorded during sessions and expanded promptly afterwards to preserve detail on workflow sequences, bottlenecks, and operational constraints.

The FGDs were used to capture shared experiences and to explore how perspectives converged or diverged across cadres involved in documentation, supervision, and implementation support. Participants included MoH staff, healthcare providers, technical and implementing partners, and managerial or administrative staff at facility, regional, and national levels, as well as researchers and senior academic staff from organisations collaborating with the government. The discussions were facilitated by the lead researcher (ON, a female social scientist) and by trained members of the research team, using a semi-structured guide (S3 Text) informed by findings from interviews and participant observations. The discussions were designed to encourage participation across different cadres of staff and were held in settings that ensured privacy and minimal interruptions. Each FGD lasted between 1 hour 45 minutes and 2 hours 45 minutes, with an average duration of two hours. The discussions were audio-recorded and transcribed, and facilitators maintained structured notes on group dynamics, points of consensus, disagreement, and issues requiring follow-up during subsequent interviews or observations.

Across all methods, the research team held weekly debriefings to synthesise emerging insights, identify areas requiring clarification, and refine prompts for subsequent data collection. This iterative process supported RAP's purpose of producing timely, implementation-relevant learning while maintaining methodological transparency and coherence across the dataset.

## Research team and reflexivity

The multidisciplinary research team included social scientists, public health researchers, and trained field assistants with expertise in qualitative research, maternal and newborn health, health systems, and digital health implementation. The lead researcher, ON, is a senior social scientist with an MPH and extensive experience in qualitative and implementation research and worked closely with the broader team to oversee data collection and analysis. Before the qualitative study began, the team had an established working relationship with the Ministry of Health and was already familiar with the wider SPT initiative. Several team members also had prior experience in maternal and child health research in The Gambia, as well as detailed knowledge of the health system, routine documentation practices, and the sociocultural context, all of which strengthened both data collection and interpretation.

Because SPT was a ministry-led pilot integrated into routine services, we anticipated that some participants, especially facility staff, might feel pressured to provide only “positive” answers or avoid criticism. To mitigate this, we emphasised confidentiality and the voluntary nature of participation, clarified that their involvement would not affect their employment or access to support, and, whenever possible, held interviews and discussions in private spaces.

Reflexive notes were maintained throughout fieldwork, and regular team debriefings were used to consider how researchers’ roles, assumptions, and participants’ expectations might shape questioning, responses, and interpretation, and to adjust prompts to elicit both perceived benefits and implementation challenges.

## Data management and analysis

To prepare the data for analysis, all audio recordings were transcribed verbatim, and any personal information was removed to ensure participant anonymity. Observation notes were also de-identified. All files were stored on password-protected institutional computers accessible only to the research team. In the final report, participant quotes were attributed to broad stakeholder role categories (e.g., frontline healthcare provider, facility manager, health information staff, MoH official, or technical advisor) rather than specific job titles or institutions, to protect participant confidentiality while retaining relevant contextual information.

We analysed the data thematically using NVivo 14 software. First, the team read through the transcripts and field notes to gain an in-depth understanding of participants’ experiences. We then developed a coding framework based on the study objectives and issues emerging from the data. To enhance analytical rigour, we compared findings across different methods, for example, contrasting interview accounts with observational data, and across different participant groups. We paid close attention to views that differed from the majority to ensure a balanced interpretation. Finally, we grouped codes into broader themes that described how well SPT worked in practice, the challenges staff faced, and what would be needed to support successful national scale-up.

## Ethics

Ethical approval was obtained from the Gambia Government/MRCG Joint Ethics Committee (Ref: 31096) and the Observational/Interventions Research Ethics Committee of LSHTM (Ref: 31096). All interview and FGD participants received study information and provided

written informed consent prior to participation. For non-participant observations, we obtained permission from facility and unit heads. Additionally, researchers verbally informed staff and patients of their presence and the purpose of the observation, ensuring that any individual could decline to be observed without providing a reason.

### Inclusivity in global research

Additional information regarding the ethical, cultural, and scientific considerations specific to inclusivity in global research is included in the Supporting Information (S4 Text)

## Results

### Participants and data sources

A total of 30 semi-structured interviews were conducted with frontline healthcare providers, supervisors, and other stakeholders involved in implementation and routine use of SPT in maternal and infant child health services (Table 1). In addition, four FGDs were conducted with 30 participants, including MoH staff and other key stakeholders (Table 2). To complement these perspectives, structured observations were carried out across key service delivery areas involved in the pilot.

Table 1: Composition of participants who were interviewed at the facility level

| Designation | Frequency (N=30) | Percentage (%) |
|---|---|---|
| Facility-level clinical staff | 22 | 73 |
| Facility-level administrative and records staff | 5 | 17 |
| Facility-level management staff | 3 | 10 |

Table 2: Composition of participants who participated in focus-group discussions

| Designation | Frequency (N=30) | Percentage (%) |
|---|---|---|
| Ministry of Health officials | 13 | 43 |
| Technical/implementing partners | 8 | 27 |
| Facility-level clinical staff | 6 | 20 |
| Data/information system staff | 2 | 7 |
| Facility-level management staff | 1 | 3 |

## Overview of findings

Figure 1 presents the findings as a RAP-informed implementation pathway, showing how triangulated evidence and iterative feedback informed interpretation of early implementation experiences and implications for scale-up. The themes describe: (i) how SPT was co-produced and embedded into routine maternal, newborn, and child health documentation, (ii) operational and system constraints affecting implementation, (iii) perceived value and drivers of acceptance among staff, and (iv) conditions required for sustainability and national scale-up. Together, these themes reflect both the practical feasibility of implementation and the factors shaping staff acceptance of SPT in routine care.

**Figure 1: RAP-informed pathway of early SPT implementation in maternal and newborn health services in The Gambia.** *This figure presents the qualitative findings as a RAP-informed implementation pathway. It comprises four stages that summarise the main themes identified from triangulated interviews, focus group discussions, and structured observations. The feedback arrow illustrates the iterative adaptation that occurred during implementation, while the lower panel depicts key features of the Rapid Assessment Procedures (RAP) approach, including triangulation, iterative learning, and action-oriented outputs. The "Implications for scale-up" box summarises practical lessons derived from the analysis*

## 1. SPT introduction and adaptation

### 1.1 Co-produced introduction and integration

SPT was framed by participants as a co-produced intervention rather than a fixed "technology drop-in." Frontline staff described early engagement as meaningful because it enabled the system to reflect clinical realities and created a basis for legitimacy at the point of use. This sense of contribution was expressed directly by health workers:

> *"Yes, yes, yes, a lot of contributions came from us (health workers). We were asked to share our opinion... which formed part of the development." (Facility-level management staff)*

Stakeholders explained that SPT was based on existing documentation tools and improved through feedback from health workers. As a result, staff viewed it as a familiar system adapted to their routine work, rather than a completely new tool imposed from outside:

> *"The materials used were initially taken from the existing tools, the HMIS (Health Management information System) tools, and then, after certain verifications, the health workers were the ones who made contributions on what they would like to work with in their facility." (Facility-level administrative and records staff)*

However, participants also noted that participatory design did not resolve all existing weaknesses. Rather, it highlighted important omissions in the forms, especially where key maternal procedures were missing. These gaps raised concerns about clinical completeness and required further revision.

> *"All other surgeries like hysterectomy, myomectomy, and evacuation were not captured in the SPT. It was a concern I raised with the SPT team, and said they will develop forms for those also, or incorporate those surgeries inside the forms." (Facility-level clinical staff)*

Frontline accounts suggested that early mismatches between SPT variables and established registers created uncertainty about "which source is correct," particularly during the transition period when staff were still calibrating the new system against longstanding documentation routines:

> *"So, there was a push and pull, and some of the variables were not added. Not all the variables that are in the book are on the SPT." (Facility-level clinical staff)*

Overall, these accounts suggest that SPT integration was a gradual process in which staff initially had to navigate both existing documentation practices and the new forms. As gaps became apparent, feedback from routine users informed revisions to improve usability and clinical completeness.

### 1.2 Training and support as mechanisms for adoption

Training functioned as a key mechanism shaping early implementation quality. Where training was structured and practical, staff described it as building procedural confidence through repeated guided practice, not just orientation. One participant highlighted the intensity and "learning-by-doing" format:

> *"Before we started using the technology, we sometimes spent … hours training on SPT. The developer would guide us through various ... forms... Everyone [was] assigned to fill in the various forms individually, with a screen display to guide and direct us in the process…" (Facility-level administrative and records staff)*

In contrast, participants at one facility described uneven training coverage due to staff absences, which required informal workarounds:

*"Yes, there was training before the start, but not all of us were trained… Some were on leave, others travelled, so when we started it, we had a little bit of a problem, and I can say we still have some of those problems." (Facility-level clinical staff)*

These gaps shifted learning responsibility from formal training structures to colleagues and supervisors, resulting in peer-led "micro-training" that helped sustain implementation but also risked variability in practice across cadres and units:

*"No! There was no organised training from the hospital… But our senior colleagues trained us in their own local ways to make us understand it." (Facility-level clinical staff)*

Field documentation similarly indicated that cross-site learning and peer support were mobilised deliberately to minimise service disruption rather than waiting for ideal training conditions:

*"The first hospital that adopted the SPT was very involved in supporting and training the second health facility, creating innovative ways to minimise disruption to routine hospital activities." (Field notes, researcher #2)*

Participants also described remote support structures (e.g., WhatsApp) as a pragmatic extension of training and troubleshooting, particularly where routine clinical workload made in-person sessions difficult to coordinate:

*"Sometimes, we also do online group training. We created an account and a WhatsApp group, where we also receive training occasionally. Because it is normally difficult for everyone to be present at the training ground, we decided to introduce a WhatsApp training group to explain to us." (Facility-level administrative and records staff)*

### 1.3 Early use and adjustment over time

Early use was characterised by a transition period in which uncertainty about feasibility and sustainability shaped staff responses, particularly where documentation expectations were already high. Accounts suggested that initial difficulty was not simply resistance to change, but a predictable adjustment as staff learned a new precision-based form-filling process:

*"By the time they said SPT is coming, all the staff were afraid." (Facility-level clinical staff)*

> *"The start was very complicated… It was hard for the first month, but as things went on, it became easier." (Facility-level administrative and records staff)*

Concerns also extended to whether SPT would last long enough to justify the effort of adaptation:

> *"My initial thought was like… is it actually going to work? Like, is it actually going to continue, or will they withdraw it later?" (Facility-level clinical staff)*

Observations during early implementation helped contextualise these perceptions by showing how documentation competed with patient-facing work under real clinic pressures:

> *"Staff frequently struggled to balance patient care with the additional documentation time required for SPT. In some clinics, forms piled up and were completed in batches at the end of the day." (Field notes, researcher #1, antenatal clinic)*

Practical usability issues, such as limited writing space and the need to repeat entries after mistakes, increased early frustration and slowed documentation:

> *"So, the initial challenge that I encountered was that the boxes were very small. Sometimes you would start to enter up until the last point, and you'll make a mistake. So, you will have to cancel all that and start again, and that was very tedious." (Facility-level clinical staff)*

Over time, as users became more familiar with the process, confidence and efficiency improved, and many health workers described the system as easier to use than they had expected once they adapted to it.

## 2. Implementation challenges

### 2.1 Infrastructure, connectivity, and data governance issues

Across both pilot sites, infrastructure constraints shaped whether SPT functioned as a time-saving system or an additional burden. Unreliable internet connectivity was repeatedly described as the most disruptive barrier, undermining timely syncing and creating uncertainty about whether records had been successfully captured:

> *"The only problem we have is a network [internet] problem. If you register a person today, before it gets updated, it [could] take [up to] two months sometimes." (Facility-level administrative and records staff)*

> *"Yes! The network [internet] is always a problem. Everybody knows the network here... At times, you will have to register the person again, which is not the aim of the project." (Facility-level administrative and records staff)*

When syncing delays occurred, staff described being unable to retrieve information when needed, reinforcing perceptions that system performance was dependent on factors outside frontline control:

> *"Some of the challenges are with the records [department]. When you need information or data... they will tell you that the data is not yet synced, or at times they will tell you that there is no internet connection." (Facility-level clinical staff)*

Participants also noted that storing completed paper forms after scanning could become difficult as record volumes increased, especially where secure storage space was limited.

> *"We also need more space, so that any document we scan can be kept safe. There is space, but it is not enough." (Facility-level administrative and records staff)*

Beyond connectivity and storage space, higher-level stakeholders also raised concerns about data governance. In particular, they emphasised that long-term sustainability would require alignment with national expectations around data hosting, server infrastructure, and data protection.

> *"The infrastructure needs to be on the Gambian servers because if not... there will be lots of questions and you will even have to face lots of challenges… because of these data protection laws." (Ministry of Health officials)*

### 2.2 Data capture challenges

Participants also described data capture challenges related to how forms were completed and read by the scanner. Problems such as illegible handwriting, faint ink, or writing outside the required spaces sometimes meant that forms could not be read accurately and had to be corrected or re-entered:

> *"With the SPT, you are constrained. There is a margin that you should not go beyond. So, you need to stop there... Some people's handwriting is not legible enough for the computer to read it. I think that's a challenge." (Facility-level clinical staff)*

> *"During the first month of implementation, several staff members were observed rewriting forms that the scanner could not read due to faint ink or slightly misaligned*

*writing. In one clinic, staff kept "correction folders" where they stored forms that needed to be re-entered later." (Field notes, researcher #2, antenatal clinic).*

Where variable duplication existed across forms, staff interpreted this as inefficient design that increased effort without clear added value, particularly when the same information was captured in multiple places:

*"One quick challenge ... is that there are duplications of variables in certain SPT forms. For example, looking at the antenatal form, you have lab variables there, and in the lab form, you also have the same variables." (Facility-level clinical staff)*

In addition, some staff reported discrepancies between paper entries and system-generated outputs after scanning, raising concerns about reliability and trust in automated reporting, especially because routine reporting depends on the perceived correctness of aggregated figures:

*"The SPT helps to generate the reports, but the challenge we have is that... there are some inconsistencies when it comes to numbers, on the paper and the report on the system." (Facility-level clinical staff)*

### 2.3 Workload pressures and workflow adjustments

A major challenge during implementation was the period of dual documentation. Staff described this as increasing their workload, reducing time for patient care, and pushing documentation into after-hours work. Although this was intended as a temporary transition, its continuation reduced acceptance of SPT because staff could not yet experience the system as saving time or improving efficiency:

*"We are doing the SPT and again recording in our registers just like how we were doing it before, with the SPT now adding another problem for us." (Facility-level clinical staff)*

This burden was particularly salient in high-volume units and during long shifts, where the "end of shift" did not equate to completion of documentation obligations:

*"The reason why I said that it is time-consuming is that after closing from a night shift working for 12 hours tirelessly, from 20:00 to 08:00, and before going home, you must complete these forms depending on the number of deliveries you have conducted." (Facility-level clinical staff)*

*"In both hospitals, documentation often continued after patient care hours, and staff were seen dividing tasks among themselves, one providing care, another catching up on SPT entries." (Field notes, researcher #1, labour ward)*

Overall, the workflow burden was uneven and most acute in high-volume, understaffed units. Under these conditions, completing SPT documentation after clinical care became time-consuming, indicating that successful integration depends on adequate staffing and task-sharing.

### 3. Perceived benefits and drivers of acceptability

#### 3.1 Cost and continuity of care

Despite early barriers, participants increasingly evaluated SPT against the costs of paper registers and the strategic aspiration to move toward digitisation. Some stakeholders framed SPT as a financially realistic reform in a resource-constrained system, describing it as a mechanism to reduce long-term printing and operational costs while improving data integrity:

*"To me, this will have a transformative impact since it is cost-effective, and we all know that the financing when it comes to health is very low… This will minimise a lot of costs in almost all the health facilities in the region, and it will also help in data completeness and data integrity." (Facility-level management staff)*

Drawing on earlier national experience with SPT in immunisation services, one respondent contrasted previous printing costs with the lower costs of running SPT:

*"During the national evaluation for the Expanded Programme on Immunisation component, the annual printing cost was higher. However, with SPT, the cost is now less… So, it is far cheaper to operationalise SPT than the conventional printing cost." (Ministry of Health officials)*

Participants described the linkage of maternal and infant records as a practical advantage because it made patients easier to trace, reduced the risk of lost identifiers, and supported follow-up and continuity of care:

*"One of the advantages is that the mother's and child's numbers are linked. If a child loses their card and the mother is registered, you can access the mother's SPT record and find the linked child's number." (Facility-level clinical staff)*

### 3.2 Efficiency and data quality gains

Once staff became more familiar with SPT, its efficiency benefits became clearer. Participants highlighted faster retrieval of information, less dependence on paper records that could be lost or damaged, and easier reporting. In contrast, paper registers were described as more fragile and less accessible:

> *"Because the SPT is in the system, with just a click, you will get access to whatever you want to check. But the books can go missing, they can tear off, and they can be misplaced". (Facility-level clinical staff)*

Some participants described speed as both an efficiency outcome and a marker of adaptation, particularly for high-volume documentation tasks:

> *"With the SPT, you can record more than 30 prescription pads within an hour, making it easier. It is faster, and it requires less time, compared to the register; it is very different". (Facility-level administrative and records staff)*

Automated reporting was also described as shifting routine data work away from manual tallying, with implications for timeliness and accountability in service reporting.

### 3.3 Acceptance of SPT in daily practice

Staff acceptance and routine use of SPT grew as confidence increased and support remained available. Participants suggested that hands-on experience, peer support, and troubleshooting helped staff feel more able to use the system in daily practice. Over time, SPT became less of an unfamiliar extra task and more a part of routine work.

Participants described staff support for SPT in relation to the practical advantages it offered in daily work, particularly easier reporting and quicker access to patient information.

> ***"****Before, we used to prepare statistics on a weekly or monthly basis for each condition. SPT has made it easier for us by the end of every month. With SPT, these are automatically generated and segregated. SPT has made life easier for us; that's the reason why we like using it." (Facility-level administrative and records staff)*

Beyond efficiency, participants highlighted the clinical usefulness of linked maternal and infant records. They explained that access to connected information supported follow-up, continuity of care, and decision-making during emergencies or later visits:

*"If you have the correct SPT number, you can see the woman's antenatal visits, when and where she delivered, and the baby's information after delivery." (Facility-level clinical staff)*

Our observations also showed that, as staff became more familiar with the system, they used it more confidently. This suggested a shift from early uncertainty to greater ease with SPT.

## 4. Sustainability and prospects for scale-up

### 4.1 Policy and strategic alignment

Stakeholders described scale-up as both an operational and a governance issue. They emphasised that high-level engagement was needed to move SPT from a pilot project to an institutional commitment, with clear mandates, funding, and system-wide communication:

*"Stakeholder engagement should be done at a very high level and there should be certain critical decisions to be taken or commitments to be made by the Ministry of Health and the government to ensure that this will continue". (Facility-level clinical staff)*

Participants viewed policy alignment as supportive of SPT implementation. They noted that SPT was consistent with broader government efforts to strengthen digital health systems and improve routine health information management. However, they also emphasised that policy support alone would not be enough without the infrastructure, training, and technical support needed for implementation:

*"Currently, I think the policy of the Ministry is in line with the SPT… policy-wise, I think the policies are in favour of the SPT." (Ministry of Health officials)*

### 4.2 Human resource, training continuity, and local technical capacity

SPT did not remove the need for data work; rather, it redistributed it and made certain roles, such as scanning, verification, synchronisation, and troubleshooting, more important. Shortages in these areas were seen as a direct threat to sustainability:

*"We need to have human resources. The data managers are not there. And the necessary equipment needs to be there." (Ministry of Health officials)*

*"[Take] consideration of [an] adequate number of data entry clerks… we need the right resources there." (Ministry of Health officials)*

Ongoing capacity-building was also seen as essential for replication and system stability, particularly if SPT is expanded to facilities with less implementation support:

> *"There should always be continuous training of workers who can replicate the SPT system." (Data/information system staff)*

> *"Quarterly training… about four to five trainings in a year… [would] actually help in mastering SPT." (Facility-level clinical staff)*

There was also emphasis on strengthening local problem-solving capacity so that technical issues could be addressed without prolonged dependence on external actors.

### 4.3 Government commitment and phased expansion

Long-term sustainability was closely linked to domestic financing and a planned transition from donor-supported piloting to government ownership. This was described as requiring early clarity on government commitment and deliberate planning for continuity as external support declines:

> *"There should be a stakeholder engagement to know the level of commitment from the Ministry of Health and the government as a whole… [and] preparation for transitioning when the project or the funding donors are about to phase out." (Facility-level clinical staff)*

Scale-up was also seen as feasible if evidence from the pilot sites was used to guide a phased approach, with attention to sensitisation and the time needed for acceptance to develop:

> *"Once we can see a very positive outcome from the two facilities as a pilot, then the government would now have to take it up… government and other partners will put in resources to maintain the system." (Ministry of Health officials)*

> *"When we want to scale to other hospitals… they need to sensitise first… because acceptance is the key challenge we always have." (Ministry of Health officials)*

Participants' accounts also suggested that peer influence could support implementation during expansion, with confident early users acting as local champions.

## Discussion

This qualitative study explored the early implementation of SPT in maternal and infant health services in two public health facilities in The Gambia. We found that SPT was viewed as

feasible and acceptable largely because it was built on familiar documentation practices, introduced through a participatory process, and offered visible advantages in reporting and record retrieval. However, feasibility was contingent on implementation conditions, including the extent and continuity of training, the availability of on-site support, the reliability of connectivity and scanning processes, and the amount of parallel documentation required. Similar implementation dynamics have been reported for hybrid paper-digital systems in other LMIC settings, where the benefits of digitisation can be undermined by workflow duplication and infrastructure constraints (22-24).

SPT's hybrid design allowed health workers to maintain a familiar paper-based workflow at the point of care while enabling digital data processing afterward. Many participants viewed this as a practical alternative to fully electronic medical records in settings where internet connectivity is unreliable and ICT capacity is limited. Because SPT aligned with existing routines, it appeared to support early uptake and reduce resistance to a new documentation system. This is consistent with evidence that digital health interventions are more likely to be adopted when they fit established work practices and minimise additional documentation burdens (25, 26). However, the usefulness of digitised outputs depended on reliable scanning, verification, and timely feedback to support data quality and user trust. Digital readiness is therefore not only a technical issue but also an organisational and workforce one, requiring investment in training, support systems, and ongoing alignment with clinical practice as systems evolve (25, 26).

Implementation challenges primarily centred on training coverage, workflow complexity, and infrastructure. While participants found the initial training and mentorship helpful, the lack of regular refresher training and uneven exposure across staff led to variable competence and confidence in using the system. Parallel documentation, particularly when SPT did not fully replace existing registers, was widely seen as increasing workload and creating opportunities for inconsistencies across records. These issues mirror those reported in other digital health rollouts where dual systems persist during transition, and implementation relies heavily on a small number of trained staff (27-29). Despite these constraints, both facilities adapted through peer mentoring, WhatsApp-based troubleshooting, and local workarounds to sustain operations. This experience highlights how adaptive capacity and local problem-solving were crucial for maintaining momentum (30, 31).

Stakeholder perspectives broadly reflected participants' positions within the implementation pathway. Frontline staff focused on ease of use, time pressures, and the operational implications of scanning delays and duplicate documentation (32). In contrast, managers and policymakers emphasised data completeness, accountability, and the potential of SPT to strengthen routine reporting and decision-making. This pattern is consistent with wider digital health literature indicating that end-users tend to prioritise workflow implications, whereas higher-level actors focus more on information outputs and system performance (33). Despite these differences, there was a broad agreement that scale-up would require strong support structures, including ongoing training, clear governance arrangements, and investments in ICT and health information systems to avoid overburdening staff and compromising data quality (34, 35).

Several practical lessons emerged. First, staff confidence depended on ongoing, facility-based training and refresher support, particularly where initial training coverage had been uneven. Second, dual documentation may be useful as a temporary transition measure, but it should be clearly time-limited to avoid prolonged workload burden. Finally, implementation is likely to be strengthened by practical supports such as adequate storage, clear standard operating procedures, and defined responsibilities for scanning, verification, and troubleshooting, together with continued user feedback to refine form content and layout.

### Strengths and limitations

As a qualitative study, we did not assess quantitative performance indicators such as documentation completeness, timeliness of data availability, concordance between paper and digital records, or error rates across scanning and verification. These outcomes are being examined in a separate evaluation, which will help determine whether the perceived benefits identified here are matched by measurable improvements in data quality and system performance.

Some of the challenges identified were also systemic rather than specific to the pilot facilities. Participants noted that SPT did not remove broader constraints such as staffing shortages, inconsistent internet connectivity, and limited ICT infrastructure. They also raised governance and regulatory concerns related to data ownership, access, and long-term stewardship, particularly where external technical partners or cross-border data processing may be involved (36-38).

A key strength of this study was the use of the RAP framework to triangulate in-depth interviews, focus groups, and structured observations across frontline, managerial, and policy levels (figure 1). This multi-source design strengthened the analytical credibility of the findings and allowed us to examine not only perceived acceptability, but also the contextual and operational conditions shaping SPT use in routine maternal and infant care. However, the findings are based on two pilot facilities and may not reflect implementation dynamics in smaller, more rural, or lower-resourced settings where staffing, infrastructure, and supervision arrangements differ.

### Conclusion

This study suggests that SPT is a feasible and broadly acceptable approach to improving routine maternal and infant health documentation in The Gambia. Its acceptability stems from its reliance on familiar paper-based workflows while still enabling downstream digitisation. However, participants stressed that these benefits were contingent on effective implementation, including sustained training and supervision, reliable scanning and connectivity, clear role allocation, and minimised parallel documentation. Overall, the findings indicate that hybrid documentation systems may offer a realistic pathway to digitisation in LMIC settings, provided they are underpinned by strong organisational systems, sufficient workforce support, and robust governance arrangements.

## Acknowledgements

The authors would like to thank the management and staff of Bundung Maternal and Child Health Hospital and Fajikunda Health Centre; the participants who took part in the discussions and interviews; the *Get Ready Get Real* research team; and the Gambia Ministry of Health for their unwavering support throughout this research.

## References

1. Joseph L, Lavis A, Greenfield S, Boban D, Jose P, Jeemon P, Manaseki-Holland S. A systematic review of home-based records in maternal and child health for improving informational continuity, health outcomes, and perceived usefulness in low and middle-income countries. PLoS One. 2022;17(8):e0267192. 10.1371/journal.pone.0267192

2. Kitila SB, Feyissa GT, Olika AK, Wordofa MA. Maternal Healthcare in Low- and Middle-Income Countries: A Scoping Review. Health Services Insights. 2022;15:11786329221100310. 10.1177/11786329221100310

3. Wojcieszek AM, Bonet M, Portela A, Althabe F, Bahl R, Chowdhary N, et al. WHO recommendations on maternal and newborn care for a positive postnatal experience: strengthening the maternal and newborn care continuum. BMJ Glob Health. 2023;8(Suppl 2). 10.1136/bmjgh-2022-010992

4. Till S, Mkhize M, Farao J, Shandu LD, Muthelo L, Coleman TL, et al. Digital Health Technologies for Maternal and Child Health in Africa and Other Low- and Middle-Income Countries: Cross-disciplinary Scoping Review With Stakeholder Consultation. J Med Internet Res. 2023;25:e42161. 10.2196/42161

5. Ahmer H, Farooqui K, Jivani K, Adamjee R, Hoodbhoy Z. Applying the principles for digital development to improve maternal and child health in the Peri-urban areas of Karachi, Pakistan. PLOS Digit Health. 2024;3(1):e0000434. 10.1371/journal.pdig.0000434

6. WHO recommendations on maternal and newborn care for a positive postnatal experience. Geneva: World Health Organization; 2022.

7. Bisrat A, Minda D, Assamnew B, Abebe B, Abegaz T. Implementation challenges and perception of care providers on Electronic Medical Records at St. Paul's and Ayder Hospitals, Ethiopia. BMC Med Inform Decis Mak. 2021;21(1):306. 10.1186/s12911-021-01670-z

8. Joseph L, Lavis A, Greenfield S, Boban D, Humphries C, Jose P, et al. Systematic review on the use of patient-held health records in low-income and middle-income countries. BMJ Open. 2021;11(9):e046965. 10.1136/bmjopen-2020-046965

9. Ali SK, Khan H, Shah J, Nadeem Ahmed K. An electronic health record system implementation in a resource limited country—lessons learned. DIGITAL HEALTH. 2023;9:20552076231203660. 10.1177/20552076231203660

10. Jimma BL, Enyew DB. Barriers to the acceptance of electronic medical records from the perspective of physicians and nurses:A scoping review. Informatics in Medicine Unlocked. 2022;31:100991. https://doi.org/10.1016/j.imu.2022.100991

11. Kamanga A, Ngosa L, Aladesanmi O, Zulu M, McCarthy E, Choba K, et al. Reducing maternal and neonatal mortality through integrated and sustainability-focused programming in Zambia. PLOS Glob Public Health. 2022;2(12):e0001162. 10.1371/journal.pgph.0001162

12. Molenaar J, Beňová L, Christou A, Lange IL, van Olmen J. Travelling numbers and broken loops: A qualitative systematic review on collecting and reporting maternal and neonatal health data in low-and lower-middle income countries. SSM - Population Health. 2024;26:101668. https://doi.org/10.1016/j.ssmph.2024.101668

13. Unkels R, Manzi F, Kapologwe NA, Baker U, Ahmad A, Nabiev R, et al. Feasibility, usability and acceptability of a novel digital hybrid-system for reporting of routine maternal health information in Southern Tanzania: A mixed-methods study. PLOS Glob Public Health. 2023;3(1):e0000972. 10.1371/journal.pgph.0000972

14. Äijö A, Schäffner I, Waiswa P, Kananura RM, Tessma MK, Hanson C. Assessment of a novel scanner-supported system for processing of child health and immunization data in Uganda. BMC Health Services Research. 2020;20(1):367. 10.1186/s12913-020-05242-1

15. Africa WROf. Smart Paper Technology (SPT) Kenya: © 2024 WHO | Regional Office for Africa; 2024 [Available from: https://innov.afro.who.int/page/107-smart-paper-technology-spt.

16. Strassburger S. Smart Paper Technology adoption at scale in Western Regions, The Gambia: Shifo Foundation; 2020 [Available from: https://medium.com/shifo-news/smart-paper-technology-adoption-at-scale-in-western-regions-the-gambia-29f603c2a86f.

17. Times TA. The Gambia launches digital immunization registry: Global Voices; 2022 [Available from: https://globalvoices.org/2022/07/06/the-gambia-launches-digital-immunization-registry/.

18. Sowe A, Gariboldi MI. An assessment of the quality of vaccination data produced through smart paper technology in The Gambia. Vaccine. 2020;38(42):6618-26. https://doi.org/10.1016/j.vaccine.2020.07.074

19. Prauseová J, Nkereuwem O, Okomo U, Bittaye M, Marena M, Fofana S, et al. Toward an Electronic Pregnancy Registry in The Gambia: Linking up Maternal and Newborn Health Data Using the Smart Paper Technology. Pediatr Infect Dis J. 2025;44(2s):S119-s22. 10.1097/inf.0000000000004677

20. Holdsworth LM, Safaeinili N, Winget M, Lorenz KA, Lough M, Asch S, Malcolm E. Adapting rapid assessment procedures for implementation research using a team-based approach to analysis: a case example of patient quality and safety interventions in the ICU. Implementation Science. 2020;15(1):12. 10.1186/s13012-020-0972-5

21. Palinkas LA, Zatzick D. Rapid Assessment Procedure Informed Clinical Ethnography (RAPICE) in Pragmatic Clinical Trials of Mental Health Services Implementation: Methods and Applied Case Study. Adm Policy Ment Health. 2019;46(2):255-70. 10.1007/s10488-018-0909-3

22. Cronin M, Munishi L, Olomi GA, Mitao M, Mmbaga BT, Somi J, et al. Exploring healthcare staff experiences with a hybrid paper/digital health management information system and their perspectives on digitalization as an alternative - A Tanzanian qualitative case study on perinatal data. PLoS One. 2025;20(11):e0335644. 10.1371/journal.pone.0335644

23. Ddamba A, Nsubuga B, Kamabare M, Abaho E, Alinda K, Arinaitwe D, et al. Factors influencing the availability and use of electronic medical records systems in public health facilities in Uganda: a cross-sectional assessment. BMC Med Inform Decis Mak. 2025;25(1):372. 10.1186/s12911-025-03190-6

24. Woldemariam MT, Jimma W. Adoption of electronic health record systems to enhance the quality of healthcare in low-income countries: a systematic review. BMJ Health Care Inform. 2023;30(1). 10.1136/bmjhci-2022-100704

25. Steenkamp I, Peltonen LM, Chipps J. Digital health readiness – insights from healthcare leaders in operational management: a cross-sectional survey. BMC Health Services Research. 2025;25(1):240. 10.1186/s12913-024-12129-y

26. Raunaq FF, Islam S, Anam MZ, Bari ABMM. Assessing the challenges to digital technology adoption in the healthcare sector: Implications for sustainability in emerging economies. Informatics and Health. 2025;2(2):194-209. https://doi.org/10.1016/j.infoh.2025.09.001

27. Meghana RVNSP, Singh J, Huda RK. Challenges, barriers, and facilitators in implementing digital health initiatives for NP-NCD management in India's public health system: A scoping review. Clinical Epidemiology and Global Health. 2025;35. 10.1016/j.cegh.2025.102137

28. Alhaji MM, Balarabe MR, Atama D, Okafor A, Solana D, Meyo F, et al. Motivators and barriers to the uptake of digital health platforms for family planning services in Lagos, Nigeria: A mixed-methods study. DIGITAL HEALTH. 2025;11:20552076251349624. 10.1177/20552076251349624

29. Yew SQ, Trivedi D, Adanan NIH, Chew BH. Facilitators and Barriers to the Implementation of Digital Health Technologies in Hospital Settings in Lower- and Middle-Income Countries Since the Onset of the COVID-19 Pandemic: Scoping Review. J Med Internet Res. 2025;27:e63482. 10.2196/63482

30. Bekele TA, Gezie LD, Willems H, Metzger J, Abere B, Seyoum B, et al. Barriers and facilitators of the electronic medical record adoption among healthcare providers in Addis Ababa, Ethiopia. Digit Health. 2024;10:20552076241301946. 10.1177/20552076241301946

31. Mensah NK, Adzakpah G, Kissi J, Abdulai K, Taylor-Abdulai H, Johnson SB, et al. Health professionals' perceptions of electronic health records system: a mixed method study in Ghana. BMC Med Inform Decis Mak. 2024;24(1):254. 10.1186/s12911-024-02672-3

32. Jeilani A, Hussein A. Impact of digital health technologies adoption on healthcare workers' performance and workload: perspective with DOI and TOE models. BMC Health Services Research. 2025;25(1):271. 10.1186/s12913-025-12414-4

33. Lee S, Lee YJ, Kim S, Choi W, Jeong Y, Rhim NJ, et al. Perceptions on Data Quality, Use, and Management Following the Adoption of Tablet-Based Electronic Health Records: Results from a Pre-Post Survey with District Health Officers in Ghana. J Multidiscip Healthc. 2022;15:1457-68. 10.2147/jmdh.S368704

34. Dolan SB, Alao ME, Mwansa FD, Lymo DC, Bulula N, Carnahan E, et al. Perceptions of factors influencing the introduction and adoption of electronic immunization registries in Tanzania and Zambia: a mixed methods study. Implementation Science Communications. 2020;1(1):38. 10.1186/s43058-020-00022-8

35. Craig A, Lawford H, Miller M, Chen-Cao L, Woods L, Liaw ST, Godinho MA. Use of Technology to Support Health Care Providers Delivering Care in Low- and Lower-Middle-Income Countries: Systematic Umbrella Review. J Med Internet Res. 2025;27:e66288. 10.2196/66288

36. Ogbodo DC, Awan I-U, Cullen A, Zahrah F. From Regulation to Reality: A Framework to Bridge the Gap in Digital Health Data Protection. Electronics. 2025;14(13):2629.

37. Munung NS, Staunton C, Mazibuko O, Wall PJ, Wonkam A. Data protection legislation in Africa and pathways for enhancing compliance in big data health research. Health Research Policy and Systems. 2024;22(1):145. 10.1186/s12961-024-01230-7

38. Holly L, Thom S, Elzemety M, Murage B, Mathieson K, Iñigo Petralanda MI. Strengthening health data governance: new equity and rights-based principles. International Journal of Health Governance. 2023;28(3):225-37. 10.1108/ijhg-11-2022-0104

## Supporting information

S1 Table. COREQ checklist

S1 Text. Interview topic guide

S2 Text. Interview guide

S3 Text. Focus group discussion guide

S4 Text. Inclusivity in global research

## Ethics approval and consent for publication

Ethical approval for the qualitative study was obtained from The Gambia Government/MRC Joint Ethics Committee (Reference 31096) and the Observational/Interventions Research Ethics Committee of LSHTM (Ref: 31096).

Figure 1

Click here to access/download;Figure;Figure 1.tif

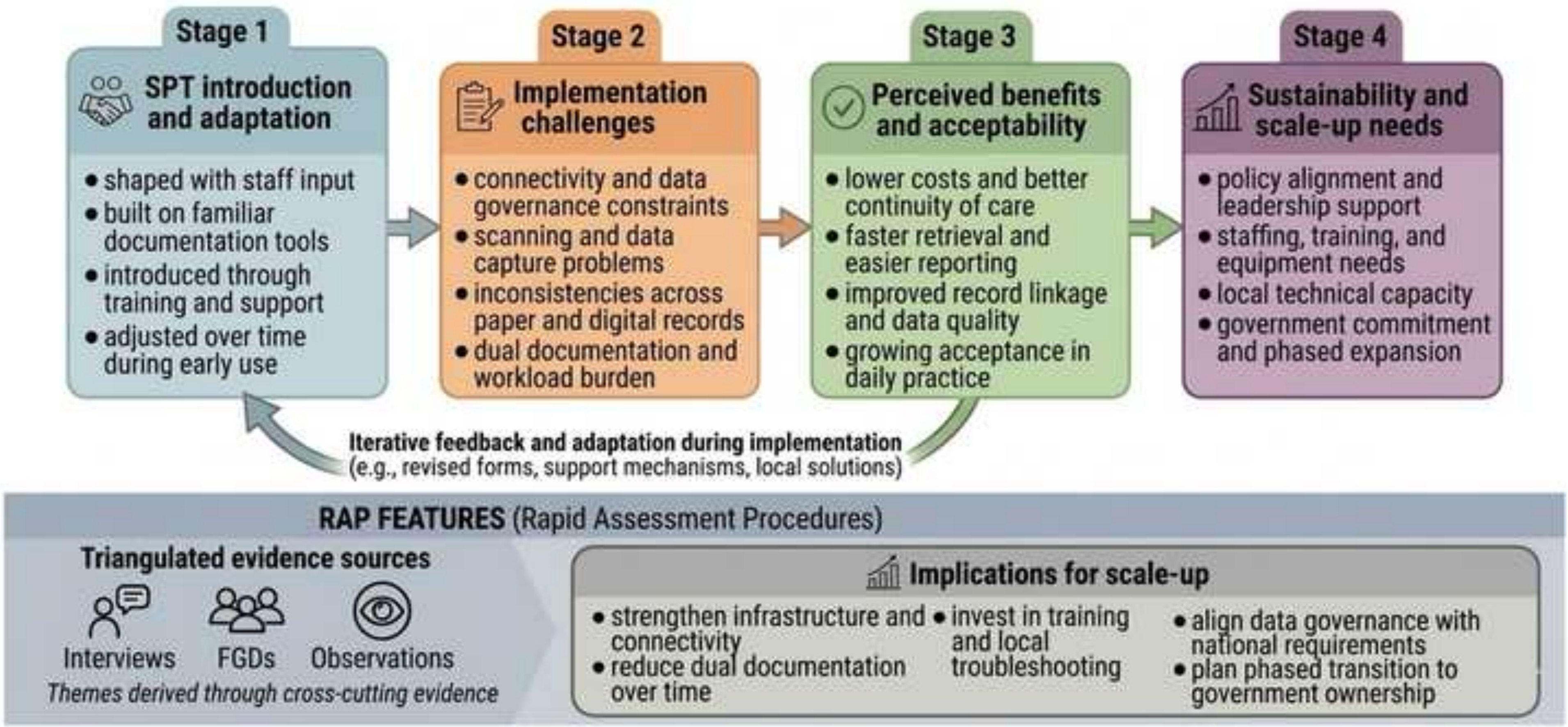

S1 Table. COREQ checklist

Click here to access/download
**Supporting Information**
S1 Table. COREQ checklist.pdf

Click here to access/download
**Supporting Information**
S1 Text. Interview topic guide.docx

Click here to access/download
**Supporting Information**
S2 Text. Observation guide.docx

Click here to access/download
**Supporting Information**
S3 Text. Focus group discussion guide.docx

Click here to access/download
**Supporting Information**
S4 Text. Inclusivity in global research questionnaire.pdf
