# Supplementary material for: Smart Paper Technology for maternal and newborn health data in The Gambia: A qualitative study of early implementation
Source: PLOS Glob Public Health. 2026 Oct 23;6(10):e0006244. doi: 10.1371/journal.pgph.0006244 (PMC13693910; doi:10.1371/journal.pgph.0006244)
Supplement: Supplementary file 6 [file pgph.0006244.s004.docx]

# **SPT Evaluation Interview Guide Version 1.0**

- Date: [Insert Date]
- Start time: [Insert Time]
- End time: [Insert Time]
- Location: [Specify Location]
- Participant ID: [To be assigned]
- Audio file ID: [To be assigned]
- Name of Interviewer: [Insert Interviewer’s Name]
- Name of Interpreter (if applicable): [Insert Interpreter’s Name]

**Purpose of the Interview:**

This guide is designed to facilitate structured interviews with healthcare workers using Smart Paper Technology (SPT) in maternal and newborn health service settings in The Gambia. The goal is to gather in-depth insights on SPT's integration, adaptation, and effectiveness in improving data management and healthcare delivery.

**Introduction (to be read by the interviewer):**

Good [morning/afternoon], my name is [Interviewer’s Name] from MRCGatLSHTM. Today’s date is [Insert Date], and the time is [Insert Time]. As the participation information sheet explains, we are here at [Location] to discuss your experiences implementing Smart Paper Technology in your work setting, program office and the Ministry of Health.

Thank you for agreeing to participate. Your insights are crucial to our research.

This interview will be recorded to capture your responses accurately, but only with your consent. I want to assure you that all information you provide will remain confidential and be anonymised in any publications or reports from this study. Your privacy and the security of your data are our utmost priority. Before we start, do you have any questions, or is there anything you need clarified?

**Interview Questions:**

**User Experience and Interface:**

1. Can you describe when and how Smart Paper Technology was introduced to your facility? *(Probe: Could you walk me through your first day using SPT? What were your initial thoughts and feelings about this new technology?)*
2. How do you and other healthcare staff describe their experience with SPT regarding design, ease of use, and overall user satisfaction? (*Probe: Are there specific features of the design that you and other users find beneficial or challenging? What improvements would you suggest?)*
3. What training or support was provided to help you adapt to using SPT? *(Probe: Can you tell me about a moment during the training that stood out to you? How did this moment impact your comfort or proficiency with SPT?)*

**Organisational Adaptation**

4. How has SPT been integrated into your daily workflow? *(Probe: Could you describe a day when SPT made a difference in your workflow? What changes have you noticed in your daily tasks since integrating SPT?* Can you describe a typical scenario where you use SPT and its impact on that task? (*Probe: Can you share a story where SPT significantly impacted your handling of a patient’s case? What would have been different without SPT?)*
5. How well has SPT adapted to fit your healthcare setting? (*Probe: What personal or organisational challenges have been encountered, and how have they been addressed?)*

**Evaluation of SPT Impact**

6. In your opinion, how has SPT impacted the quality of data management regarding accuracy and completeness? *(Probe: Can you think of a specific instance where SPT directly impacts the accuracy or completeness of the data you were handling?)*
7. Have you noticed any changes in the efficiency or quality of healthcare delivery since the introduction of SPT in your department/hospital? *(Probe: Can you describe a situation where SPT directly influenced the outcome of healthcare delivery? What do you think would have happened in this case without SPT?)*
8. How has SPT affected collaboration and data sharing between different departments within this healthcare facility? *(Probe: Are there noticeable improvements in how data is shared and utilised for patient care planning and delivery?)*

**Challenges and Facilitators**

9. Have any particular aspects of SPT facilitated your work or made tasks easier? (*Probe: Can you describe an experience where SPT made your work significantly easier? How did this influence your feelings towards using technology in healthcare?)*
10. What challenges have you encountered while using SPT? (*Probe: Can you describe a particularly challenging day with SPT? What happened, and how did you handle the situation?)*

**Future Prospects and Recommendations**

11. Based on your experience, how would you describe using the SPT in the long term within your facility? (*Probe: Looking into the future, how do you see SPT fitting into the long-term goals of your department or facility? What would need to change to make it a permanent solution?)*
12. What improvements or additional features would you suggest to enhance the functionality and effectiveness of SPT? *(Probe: If you had the power to enhance or add a feature to SPT, what would it be and why? In what way do you think this would improve your work or patient outcomes?)*
13. What advice would you give to a facility with a similar setting as your facility planning to roll out SPT as a replacement for paper-based data collection and management?

*(Can you think of any specific preparations or changes that should be made in the facility before introducing SPT to ensure a smoother transition from paper-based systems? From your experience, are there particular challenges you think a new facility should anticipate and plan for when implementing SPT?)*

**Conclusion (by the interviewer):**

Thank you for sharing your valuable insights. Your participation is crucial for understanding the impacts of Smart Paper Technology and guiding its further development. Is there anything else you want to add or thoughts you wish to share?
