# Supplementary material for: Smart Paper Technology for maternal and newborn health data in The Gambia: A qualitative study of early implementation
Source: PLOS Glob Public Health. 2026 Oct 23;6(10):e0006244. doi: 10.1371/journal.pgph.0006244 (PMC13693910; doi:10.1371/journal.pgph.0006244)
Supplement: Supplementary file 7 [file pgph.0006244.s005.docx]

**Observation Guide for the Evaluation of Smart Paper Technology (SPT)**

Bundung and Fajikunda Health Facilities

**Purpose:** To describe how SPT is used in routine maternal and newborn care, how it fits into workflow, how staff and patients respond to it, and what operational or technical factors influence implementation. Record what is directly observed and heard during practice.

**1. Observation Details and Service Context**

| **Facility name** |  | **Date** |  |
| --- | --- | --- | --- |
| **Service area** | Registration / ANC booking / Routine ANC / Laboratory / Delivery / Postnatal care / Infant welfare clinic/EPI / Other: _________________________ | **Observer** |  |
| **Start time** |  | **End time** |  |
| **No. of staff present** |  | **Approx. no. of patients seen** |  |

**Briefly describe the setting, patient flow, staffing situation, pace of work, and any visible interruptions or bottlenecks.**

__________________________________________________________________________________________________________

__________________________________________________________________________________________________________

**2. Staff Engagement with SPT in Routine Practice**

Observe how staff interact with the SPT tool in everyday care. Note whether they appear familiar with the forms, whether they use them confidently, and whether documentation seems smooth or difficult in practice.

| **Do staff appear trained and familiar with the SPT tool?** | Yes / No / Partly / Unclear |
| --- | --- |
| **How comfortable do staff appear when using the tool?** | Very comfortable / Comfortable / Somewhat comfortable / Not comfortable |
| **Is information recorded accurately, and are required fields completed consistently?** | Yes / No / Partly / Unclear |

**Describe signs of confidence or uncertainty (e.g. hesitation, asking colleagues for help, skipping fields, delayed entry, cross-checking with other records).**

__________________________________________________________________________________________________________

__________________________________________________________________________________________________________

**3. Use of SPT at the Service Point Observed**

Describe how SPT is used in the service area observed. Focus on how documentation happens in practice, whether it supports or interrupts care, and whether there are gaps, delays, or inconsistencies.

| **Registration** | How are basic patient details recorded? Do patients ask questions, and are explanations given clearly? |
| --- | --- |
| **Antenatal care** | How are clinical findings, gestational details, vaccination-related information, and follow-up plans recorded? |
| **Laboratory** | How are requests and results documented, and are there delays or missing information? |
| **Delivery/postnatal care** | How are labour details, outcomes, and immediate postnatal care documented during busy periods? |
| **Infant welfare clinic/EPI** | How are immunisation details, growth monitoring, and follow-up information recorded? |

**Field notes on service point observed:**

__________________________________________________________________________________________________________

__________________________________________________________________________________________________________

**4. Workflow, Staff Roles, and Adaptation in Practice**

Observe who completes, checks, stores, or transfers SPT forms, whether roles are clear, and how SPT fits into routine workflow. Note any duplication with paper registers and any workarounds staff use to keep services moving.

| **Who is responsible for completing and managing the forms?** | Registration staff / nurse-midwife / clinician / laboratory staff / public health staff / records-data staff / other |
| --- | --- |
| **Is there evidence of duplicate documentation or workflow disruption?** | Yes / No / Partly - describe what happens in practice |

**Describe any adaptations or workarounds used by staff to make the system function in daily practice.**

__________________________________________________________________________________________________________

__________________________________________________________________________________________________________

**5. Technical, Operational, and Contextual Factors**

| **Were any technical or operational issues observed?** | Yes / No - e.g. form shortages, storage issues, delays, connectivity, scanning or support problems |
| --- | --- |
| **Was support available when problems arose?** | Yes / No / Unclear |
| **How quickly did issues appear to be addressed?** | Immediately / Within 1 day / Within 1 week / More than 1 week / Unclear |

**Describe issues related to infrastructure, form handling, storage, movement of forms, confidentiality, or technical support.**

__________________________________________________________________________________________________________

__________________________________________________________________________________________________________

**6. Staff and Patient Responses**

Where naturally observable, record how staff and patients respond to SPT in everyday care, including visible benefits, frustrations, questions, or uncertainty.

| **How do staff appear to feel about using SPT?** | Very positive / Positive / Neutral / Negative / Very negative / Mixed |
| --- | --- |
| **Do patients appear to understand how the system is being used for their records?** | Yes / No / Partly / Unclear |

**Record any spontaneous staff or patient comments about ease of use, organisation of records, workload, delays, duplication, or continuity of care.**

__________________________________________________________________________________________________________

__________________________________________________________________________________________________________

**7. Overall Summary**

| **Overall assessment of SPT use in this service area** | Highly effective / Effective / Mixed-variable / Ineffective / Highly ineffective |
| --- | --- |

**Key strengths observed:**

____________________________________________________________________________________________________________________________________________________________________________________________________________________

**Key challenges observed:**

____________________________________________________________________________________________________________________________________________________________________________________________________________________

**Immediate recommendations or follow-up points:**

____________________________________________________________________________________________________________________________________________________________________________________________________________________
