# Supplementary material for: Smart Paper Technology for maternal and newborn health data in The Gambia: A qualitative study of early implementation
Source: PLOS Glob Public Health. 2026 Oct 23;6(10):e0006244. doi: 10.1371/journal.pgph.0006244 (PMC13693910; doi:10.1371/journal.pgph.0006244)
Supplement: Supplementary file 8 [file pgph.0006244.s006.docx]

# **FOCUS GROUP DISCUSSION GUIDE FOR SPT EVALUATION STUDY**

## **Introduction**

Good morning, everyone. My name is [Facilitator’s Name], and I am accompanied by my note-taker/assistant [Assistant's Name]. I will be your facilitator for today's group discussion.

Thank you all for agreeing to participate in this important discussion. As outlined in your consent forms and information sheets, we will record this session and take detailed notes for documentation purposes. Please rest assured that all information shared today will be kept strictly confidential, and we will anonymise all personal information following transcription of the audio recordings.

Questions

1. **Integration and Daily Use of SPT**

How has SPT been introduced into the daily healthcare data collection routines in maternal and newborn health services settings in the hospitals where the tool's piloting has been carried out? *(Probe: Can you describe changes in workflow since the adoption of SPT? What improvements or complications have arisen?)*

2. **User training and support system**

How was the initial training and onboarding process conducted for SPT? What improvements would you suggest to enhance the learning experience for new users? *(Probe: Can you describe your experiences with the training sessions? What aspect was particularly helpful or one that could have been improved? What additional support would you find beneficial in mastering the use of SPT?* *Could you describe any specific training materials or sessions that were particularly effective or ineffective? )*

3. **Challenges and Facilitators**

What major challenges have you encountered while using SPT? Are there facilitators or support mechanisms that have helped you overcome these challenges? *(Probe: Can you take us through the pre-implementation phase to this point of SPT use? )*

4. **Impact on Healthcare Service Delivery**

Discuss the impact of SPT on the quality and timeliness of healthcare delivery*. (Probe: Could you provide specific instances where SPT has affected patient record outcomes, either positively or negatively?)*

5. **Financial Implications and Cost-Effectiveness:**

What are the financial implications of adopting SPT regarding costs saved versus costs incurred? *(Probe: Is SPT cost-effective compared to the previous paper-based systems, considering both short-term and long-term financial impacts?)*

6. **Technical Support and System Maintenance:**

How robust is the technical support and maintenance framework for SPT? (*Probe: What challenges exist in maintaining SPT, and how does the current support structure address these challenges?)*

7. **Integration with Existing Systems**

How well has SPT integrated with other existing health information systems in your facility? For example, the HMIS/DHIS. What have been the experiences in this integration process? (Probe: *Are there particular system features or functions that do not work well together? How have these issues affected your daily operations?)*

8. **Sustainability and Future Directions**

How would you assess the sustainability of SPT within The Gambia's healthcare system? (*Probe: What are the key factors for successfully scaling SPT to other facilities or regions? Considering the rapid advancements in healthcare technology, how can SPT remain relevant and adaptable over the next five to ten years? What innovations or enhancements would be necessary to keep it functional and beneficial for providers and patients?)*

9. **Implementation Feedback**

What strategies are put in place for implementing SPT in facilities? On your part, what needs to be put in place to integrate SPT in maternal and newborn health service settings in The Gambia? *(Probe: What improvements or changes would enhance the effectiveness of these strategies?)*

10. **Policy Support and Barriers**

In what way are current policies supporting or hindering the effective implementation of SPT? Are there policies in place to support the continuity of Smart Paper Technology (SPT) following the end of the pilot phase? *(Probe: What policies are needed to facilitate a smoother integration of SPT into existing systems?* *What specific measures or strategies are outlined in these policies to ensure SPT's sustained use and potential scaling?)*

11. **Wrap-Up and Next Steps**

Thank you for your insightful contributions throughout today's discussions. Your feedback is important in shaping the future direction of SPT implementation across The Gambia. As we conclude, let’s summarise the key points from today's sessions and discuss the next steps to improve and expand the use of SPT in our healthcare system. We also welcome any final thoughts or suggestions you may have.
