# Supplementary material for: Smart Paper Technology for maternal and newborn health data in The Gambia: A qualitative study of early implementation
Source: PLOS Glob Public Health. 2026 Oct 23;6(10):e0006244. doi: 10.1371/journal.pgph.0006244 (PMC13693910; doi:10.1371/journal.pgph.0006244)
Supplement: Supplementary file 9 [file pgph.0006244.s010.docx]

# Reviewer: 1

| **Comments to the Author** | **Response to reviewer** |
| --- | --- |
| The manuscript is well written and presents interesting findings on the use of digital health as an innovative solution to an existing challenge. | We thank the reviewer for this comment. We now provide a point-by-point response to the reviewer’s comments below. |
| **Methods Section** |  |
| - The study design requires further elaboration. Please specify the qualitative study design used. | We have revised the *Study Design and Approach* section to explicitly specify that we employed a qualitative descriptive implementation evaluation guided by Rapid Assessment Procedures (RAP). We have also expanded the section to describe how rigour and validity were ensured within this rapid design, covering four complementary practices: multi-method triangulation across interviews, focus groups, and structured observations; purposive sampling to achieve variation across participant groups and implementation roles; iterative team debriefings to verify emerging interpretations and refine data collection guides; and reflexivity maintained throughout fieldwork via reflective field notes and team discussions. We note that these practices are consistent with the consensus-based Planning for and Assessing Rigour in Rapid Qualitative Analysis (PARRQA) framework (23). |
| - While a brief description of the intervention is provided, it would help readers better understand the findings and contextualize the quotes if you either cite a published protocol paper or include it as a supplementary file. | We have cited the published protocol paper (Prauseová et al., 2025 — reference 19 in the manuscript), which describes the SPT pilot and its implementation in detail, including the maternal and infant health documentation workflow.  A sentence directing readers to this paper has been added to the Intervention Description section: "*Full details of the intervention design, pilot implementation, and the maternal and infant health documentation workflow are described in the published protocol paper (19).*" |
| - As this study focuses on implementation outcomes, it would be important to clearly define these outcomes (e.g., acceptability, feasibility) in the methods section. For example, you may consider referencing Proctor et al., who provide well-established definitions of implementation outcomes. | We thank the reviewer for this suggestion. We have revised the Introduction to explicitly define feasibility and acceptability as implementation outcomes using Proctor et al.'s framework. Feasibility is defined as the extent to which an innovation can be successfully implemented within a given context or setting, and acceptability as the perception among stakeholders that a practice or innovation is agreeable, palatable, or satisfactory. The Proctor et al. reference has been added to the reference list (reference 20). |
| - Please include a reference guiding your thematic analysis approach. Clarifying the methodological framework (e.g., what reference informs your thematic analysis) will help ensure transparency and guide the structure and level of themes presented in the results. | We agree this is an important clarification. We have added a reference to Braun and Clarke (2006) to specify the approach underpinning our thematic analysis. The revised Data Management and Analysis section now explicitly states that thematic analysis was conducted following the approach described by Braun and Clarke (2006), which guided the process of familiarisation, coding, and theme development. The Braun and Clarke reference has been added to the reference list (reference 24). |
| **Results Section** |  |
| - Regarding the presentation of quotes: it is currently unclear whether the quotes represent a diverse range of respondents. While your current approach is acceptable, it would be helpful to clarify that the quotes reflect different participants across groups. For instance, instead of “Facility-level administrative and records staff,” consider specifying identifiers such as “Facility-level administrative and records staff, IDI-03” to improve transparency. | We have revised the attribution of participant quotes in the Results section to include unique identifiers. Interview quotes are labelled using IDI identifiers (e.g., IDI-01), while FGD quotes are labelled using identifiers that indicate both the FGD and the individual speaker within that group (e.g., FGD1-06, FGD3-04, FGD4-03). Observation excerpts are labelled separately as field notes by researcher source. This enables readers to verify that quoted material reflects a diverse range of participants across data sources and participant groups. A note explaining the identifier system has been added to the Participants and data sources section of the Results. |
| Were the participants in each of the 4 FGDs at the same professional role or were all professionals combined together?  If it was a mix and if all these 5 listed designations were part of the same FGD, how did the authors ensure that the voices of facility level staff were not overshadowed by Ministry officials or technical partners? | The FGDs were mixed-group discussions designed to capture perspectives across the SPT implementation pathway, including facility-level users, managers, Ministry of Health officials, data/information system staff, and technical/implementing partners. We have clarified this in the Methods section and added that facilitators actively encouraged contributions across participant categories to reduce the risk of facility-level perspectives being overshadowed. |
| It might be better to modify Table 2 to reflect per group composition, for better context. | We have revised Table 2 to show the composition of each FGD separately. |
| **Discussion Section** |  |
| - The discussion is well articulated. However, it would be valuable to reflect on the role of costs—specifically whether they may support or hinder the feasibility and scalability of this innovation. | We thank the reviewer for this suggestion. We have added a paragraph to the Discussion section addressing the role of costs in the feasibility and scalability of SPT. Participants identified SPT as potentially cost saving over time compared with conventional paper registers, citing reduced printing costs as reported in the EPI experience. However, we also note that upfront implementation costs, including procurement of scanning hardware, staff training, connectivity infrastructure, and ongoing technical support, may present barriers to scale-up in resource-constrained settings in the absence of external or government funding. |
| - Consider including the codebook as a supplementary file to enhance transparency and rigor. | We have provided the coding framework (codebook) as Supplementary File S5 (S5 Table). It includes the codes, subthemes, and overarching themes, along with representative quotations, as recommended. A reference to this supplementary file has been added to the Data Management and Analysis section. |

# Reviewer: 2

| **Comments to the Author** | **Response to reviewer** |
| --- | --- |
| I have reviewed the manuscript, and my detailed comments and suggestions have been embedded in the PDF version of the manuscript as red highlights with accompanying comment boxes. Overall, the manuscript has been written well and has included necessary checklists. Some general comments for consideration based on overall assessment of the manuscript are below: | We thank the reviewer for this comment. We now provide a point-by-point response to the reviewer’s comments below. |
| **OBJECTIVES:**  1. The stated objectives have two components - feasibility (of the health system) and acceptability (of healthcare providers). The terms feasibility/capability of the "health system" sounds vague. The authors may want to consider revising the wording of the objectives and use consistent and uniform terminology throughout the manuscript. | We have revised the objectives to ensure consistent terminology throughout the manuscript. The objectives now read: *“Following Proctor’s framework, we defined feasibility as the extent to which a new treatment, innovation, or evidence-based practice can be successfully implemented within a given context or setting [20]. Acceptability was defined as the perception among implementation stakeholders that a given treatment, service, practice, or innovation is agreeable, palatable, or satisfactory [20].”*  We have also ensured terminological consistency across the Abstract, Introduction, Methods, and Discussion, using “feasibility” to refer to system-level implementation capacity and “acceptability” to refer to provider-level attitudes and uptake. |
| **PARTICIPANTS:** |  |
| 1. A rationale on why both interviews and focus groups were conducted with facility level staff needs to be added. | We added a sentence to the Data Collection section to clarify the rationale for using both methods with facility-level staff. Individual interviews enabled in-depth, confidential exploration of participants’ experiences, perceptions, and concerns, particularly regarding workload and acceptability. Focus group discussions facilitated examination of shared experiences and how views converged or diverged across cadres. This complementary use of methods aligns with RAP triangulation principles and strengthens the overall validity of the findings. |
| 2. There is inconsistency in description of the participants, with several different terms being used. It seems like the terms health worker, staff, and frontline are used to describe facility-level clinical staff. The terms managers are used to describe facility level management staff and policymakers to describe Ministry of Health officials. If the authors had this grouping in mind, one way to show it could be in tables 1 and 2. | We have standardised participant terminology throughout the manuscript. We now use the following consistent terms: "*facility-level clinical staff*" (including nurses, midwives, and other frontline healthcare providers), "*facility-level management staff*" (facility administrators and supervisors), "*facility-level administrative and records staff*" (health information and records officers), and "*Ministry of Health staff*" (national-level policymakers and programme managers), and “*Technical/implementing partners”*. Tables 1 and 2 have been revised to clearly present these groupings. The term "stakeholders" has been replaced with specific group labels throughout the Results section. |
| **STUDY DESIGN:**  1. Study design needs to be elaborated with focus on how rigor and validity were ensured in data collection and analysis given the rapid nature of the study. | We have expanded the Study Design and Approach section to explicitly address how rigour and validity were maintained within the RAP framework. The revised text now details: (i) triangulation across three data sources (interviews, focus groups, and structured observations); (ii) iterative team debriefings used to verify emerging interpretations and refine data collection guides; (iii) purposive sampling to ensure variation across participant groups and implementation roles; and (iv) reflexivity maintained throughout fieldwork via reflective field notes and team discussions. |
| **INTERVENTION DESCRIPTION:**  1. TIDiER guidelines suggest using the TIDieR checklist to report interventions (reference link added in the PDF comments). | We have completed the TIDieR checklist and included it as a supplementary file (S1 Text). A statement confirming adherence to the TIDieR reporting guidelines has been added to the Intervention Description section, with a reference to the supplementary checklist. The intervention description in the main text has also been reviewed and expanded to ensure that it addresses all required TIDieR elements, including the rationale, materials, procedures, provider, mode of delivery, and fidelity. |
| **ANALYSIS:**  1. Were any frameworks or models used during preparation of the interview guide, data collection, or analysis? Some implementation-specific frameworks in literature include: CFIR (Consolidated Framework for Implementation Research), EPIS (Exploration, Preparation, Implementation, Sustainment), etc. | We thank the reviewer for raising this. Our study was guided by Rapid Assessment Procedures (RAP) as the overarching methodological framework, which shaped the multi-method design, iterative data collection, and team-based analysis approach. The interview and focus group guides were informed by Proctor et al.'s implementation outcomes framework, with feasibility and acceptability serving as the conceptual anchors for data collection and thematic organisation. While we did not apply CFIR or EPIS as primary analytical frameworks, the thematic structure of our findings, covering co-production, training, infrastructure, acceptability, and scale-up, aligns broadly with the implementation domains described in such frameworks. We have not incorporated explicit framework labelling into the manuscript text, as RAP and Proctor et al. adequately describe the methodological and conceptual approach used in this study. |
| **RESULTS:** |  |
| 1. As per earlier comment, several different terms have been used to describe the participants including the term stakeholders. The general term participants has been used several times which does not give the full picture of which group of participants' views have been reported. Being as specific as possible will make it easier for the reader to understand the findings. | This has been addressed in our response to the Participants comment above. All instances of "participants" and "stakeholders" in the Results section have been replaced with specific group labels consistent with the revised Tables 1 and 2. |
| 2. While technical/implementing partners were part of the focus groups, no quotes from these participants have been presented in the manuscript. It is suggested to include some quotes from this group as well. | We have added representative quotes from technical and implementing partners in the relevant Results sub-sections, specifically in section 4.1 (Policy and Strategic Alignment) and section 4.2 (Human Resource, Training Continuity, and Local Technical Capacity), where their perspectives on governance, sustainability, and scale-up were most salient. The quotes are now attributed using the revised participant identifier system, for example "*Technical/implementing partner, FGD1-05*" and "*Technical/implementing partner, FGD1-03*". |
| **SUPPLEMENTARY MATERIAL:** 1. It is recommended to add an appendix with codes, sub-themes/categories, themes, corresponding quotes. | We have added the complete codebook as a supplementary file (S5 Table), including codes, sub-themes, overarching themes, and representative exemplar quotes for each code to enhance the transparency and reproducibility of the analysis. |
| **CHECKLIST:** Planning for and Assessing Rigor in Rapid Qualitative Analysis (PARRQA): consensus-based framework for designing, conducting and reporting 1. This might be a helpful resource to help with revisions: [https://pmc.ncbi.nlm.nih.gov/articles/PMC11468362/](https://url.uk.m.mimecastprotect.com/s/qrTHC8EVLUpAYNAhEHvUykDgc?domain=pmc.ncbi.nlm.nih.gov) | We reviewed the PARRQA framework and used it to guide our revisions to the Study Design and Data Management and Analysis sections, particularly in how we describe data quality, rigour, and validation procedures. We also added a reference to PARRQA in the Study Design section. |
