# Supplementary material for: Smart Paper Technology for maternal and newborn health data in The Gambia: A qualitative study of early implementation
Source: PLOS Glob Public Health. 2026 Oct 23;6(10):e0006244. doi: 10.1371/journal.pgph.0006244 (PMC13693910; doi:10.1371/journal.pgph.0006244)
Supplement: Peer review history

# Peer review: Decision Letter 0

**DOI:** 10.1371/journal.pgph.0006244.r001

**Authors:** Md. Alamgir Hossain

30 Apr 2026

PGPH-D-26-01044

Smart Paper Technology for maternal and newborn health data in The Gambia: a qualitative study of early implementation

PLOS Global Public Health

Dear Dr. Nkereuwem,

Thank you for submitting your manuscript to PLOS Global Public Health. After careful consideration, we feel that it has merit but does not fully meet PLOS Global Public Health’s publication criteria as it currently stands. Therefore, we invite you to submit a revised version of the manuscript that addresses the points raised during the review process.

Please submit your revised manuscript by Jun 29 2026 11:59PM. If you will need more time than this to complete your revisions, please reply to this message or contact the journal office at globalpubhealth@plos.org. When you're ready to submit your revision, log on to https://www.editorialmanager.com/pgph/ and select the 'Submissions Needing Revision' folder to locate your manuscript file.

Please include the following items when submitting your revised manuscript:

- A letter that responds to each point raised by the editor and reviewer(s). You should upload this letter as a separate file labeled 'Response to Reviewers'.
- A marked-up copy of your manuscript that highlights changes made to the original version. You should upload this as a separate file labeled 'Revised Manuscript with Track Changes'.
- An unmarked version of your revised paper without tracked changes. You should upload this as a separate file labeled 'Manuscript'.

Guidelines for resubmitting your figure files are available below the reviewer comments at the end of this letter.

As the corresponding author, your ORCID iD is verified in the submission system and will appear in the published article. PLOS supports the use of ORCID, and we encourage all coauthors to register for an ORCID iD and use it as well. Please encourage your coauthors to verify their ORCID iD within the submission system before final acceptance, as unverified ORCID iDs will not appear in the published article. *Only*  the individual author can complete the verification step; PLOS staff *cannot*  verify ORCID iDs on behalf of authors.

We look forward to receiving your revised manuscript.

Kind regards,

Md. Alamgir Hossain, M.S.S., M.P.H.

Academic Editor

PLOS Global Public Health

Journal Requirements:

1. Please include a complete copy of PLOS’ questionnaire on inclusivity in global research in your revised manuscript. Our policy for research in this area aims to improve transparency in the reporting of research performed outside of researchers’ own country or community. The policy applies to researchers who have travelled to a different country to conduct research, research with Indigenous populations or their lands, and research on cultural artefacts. The questionnaire can also be requested at the journal’s discretion for any other submissions, even if these conditions are not met.  Please find more information on the policy and a link to download a blank copy of the questionnaire here: https://journals.plos.org/globalpublichealth/s/best-practices-in-research-reporting. Please upload a completed version of your questionnaire as Supporting Information when you resubmit your manuscript.

2. Please amend your detailed Financial Disclosure statement. This is published with the article. It must therefore be completed in full sentences and contain the exact wording you wish to be published.

a. State the initials, alongside each funding source, of each author to receive each grant.

b. If any authors received a salary from any of your funders, please state which authors and which funders.

3. We ask that a manuscript source file is provided at Revision. Please upload your manuscript file as a .doc, .docx, .rtf or .tex.

4. We note that you have indicated that there are restrictions to data sharing for this study. For studies involving human research participant data or other sensitive data, we encourage authors to share de-identified or anonymized data. However, when data cannot be publicly shared for ethical reasons, we allow authors to make their data sets available upon request. For information on unacceptable data access restrictions, please see http://journals.plos.org/plosone/s/data-availability#loc-unacceptable-data-access-restrictions.

Before we proceed with your manuscript, please address the following prompts:

a) If there are ethical or legal restrictions on sharing a de-identified data set, please explain them in detail (e.g., data contain potentially identifying or sensitive patient information, data are owned by a third-party organization, etc.) and who has imposed them (e.g., a Research Ethics Committee or Institutional Review Board, etc.). Please also provide contact information for a data access committee, ethics committee, or other institutional body to which data requests may be sent.

b) If there are no restrictions, please upload the minimal anonymized data set necessary to replicate your study findings to a stable, public repository and provide us with the relevant URLs, DOIs, or accession numbers. Please see http://www.bmj.com/content/340/bmj.c181.long for guidelines on how to de-identify and prepare clinical data for publication. For a list of recommended repositories, please see https://journals.plos.org/plosone/s/recommended-repositories. You also have the option of uploading the data as Supporting Information files, but we would recommend depositing data directly to a data repository if possible.

Please update your Data Availability statement in the submission form accordingly.

5. Some material included in your submission may be copyrighted. According to PLOS’s copyright policy, authors who use figures or other material (e.g., graphics, clipart, maps) from another author or copyright holder must demonstrate or obtain permission to publish this material under the Creative Commons Attribution 4.0 International (CC BY 4.0) License used by PLOS journals. Please closely review the details of PLOS’s copyright requirements here: PLOS Licenses and Copyright. If you need to request permissions from a copyright holder, you may use PLOS’s Copyright Content Permission form.

Please respond directly to this email or email the journal office and provide any known details concerning your material’s license terms and permissions required for reuse, even if you have not yet obtained copyright permissions or are unsure of your material’s copyright compatibility.

Potential Copyright Issues:

Figure 1.tif: Please confirm whether you drew the images / clip-art within the figure panels by hand. If you did not draw the images, please provide (a) a link to the source of the images or icons and their license / terms of use; or (b) written permission from the copyright holder to publish the images or icons under our CC-BY 4.0 license. Alternatively, you may replace the images with open source alternatives. See these open source resources you may use to replace images / clip-art:

- https://commons.wikimedia.org

- https://openclipart.org/

If the reviewer comments include a recommendation to cite specific previously published works, please review and evaluate these publications to determine whether they are relevant and should be cited. There is no requirement to cite these works unless the editor has indicated otherwise.

Additional Editor Comments (if provided):

[Note: HTML markup is below. Please do not edit.]

Reviewers' comments:

Reviewer’s Responses to Questions

**Comments to the Author**

1. Does this manuscript meet PLOS Global Public Health’s publication criteria?

Reviewer #1: Partly

Reviewer #2: Yes

**********

2. Has the statistical analysis been performed appropriately and rigorously?

Reviewer #1: Yes

Reviewer #2: N/A

**********

3. Have the authors made all data underlying the findings in their manuscript fully available (please refer to the Data Availability Statement at the start of the manuscript PDF file)?

The PLOS Data policy

Reviewer #1: No

Reviewer #2: No

**********

4. Is the manuscript presented in an intelligible fashion and written in standard English?

Reviewer #1: Yes

Reviewer #2: Yes

**********

Reviewer #1: The manuscript is well written and presents interesting findings on the use of digital health as an innovative solution to an existing challenge.

Methods Section

- The study design requires further elaboration. Please specify the qualitative study design used.

- While a brief description of the intervention is provided, it would help readers better understand the findings and contextualize the quotes if you either cite a published protocol paper or include it as a supplementary file.

- As this study focuses on implementation outcomes, it would be important to clearly define these outcomes (e.g., acceptability, feasibility) in the methods section. For example, you may consider referencing Proctor et al., who provide well-established definitions of implementation outcomes.

- Please include a reference guiding your thematic analysis approach. Clarifying the methodological framework (e.g., what reference informs your thematic analysis) will help ensure transparency and guide the structure and level of themes presented in the results.

Result section

- Regarding the presentation of quotes: it is currently unclear whether the quotes represent a diverse range of respondents. While your current approach is acceptable, it would be helpful to clarify that the quotes reflect different participants across groups. For instance, instead of “Facility-level administrative and records staff,” consider specifying identifiers such as “Facility-level administrative and records staff, IDI-03” to improve transparency.

Discussion Section

- The discussion is well articulated. However, it would be valuable to reflect on the role of costs—specifically whether they may support or hinder the feasibility and scalability of this innovation.

- Consider including the codebook as a supplementary file to enhance transparency and rigor.

Reviewer #2: Dear Authors,

I have reviewed the manuscript and my detailed comments and suggestions have been embedded in the PDF version of the manuscript as red highlights with accompanying comment boxes.

Overall, the manuscript has been written well and has included necessary checklists.

Some general comments for consideration based on overall assessment of the manuscript are below:

OBJECTIVES:

1. The stated objectives have two components - feasibility (of the health system) and acceptability (of healthcare providers). The terms feasibility/capability of the "health system" sounds vague. The authors may want to consider revising the wording of the objectives and use consistent and uniform terminology throughout the manuscript.

PARTICIPANTS:

1. A rationale on why both interviews and focus groups were conducted with facility level staff needs to be added.

2. There is inconsistency in description of the participants, with several different terms being used. It seems like the terms health worker, staff, and frontline are used to describe facility-level clinical staff. The terms managers are used to describe facility level management staff and policymakers to describe Ministry of Health officials. If the authors had this grouping in mind, one way to show it could be in tables 1 and 2.

STUDY DESIGN

1. Study design needs to be elaborated with focus on how rigor and validity were ensured in data collection and analysis given the rapid nature of the study.

INTERVENTION DESCRIPTION:

1. TIDiER guidelines suggest using the TIDieR checklist to report interventions (reference link added in the PDF comments).

ANALYSIS:

1. Were any frameworks or models used during preparation of the interview guide, data collection, or analysis?

Some implementation-specific frameworks in literature include: CFIR (Consolidated Framework for Implementation Research), EPIS (Exploration, Preparation, Implementation, Sustainment), etc.

RESULTS:

1. As per earlier comment, several different terms have been used to describe the participants including the term stakeholders. The general term participants has been used several times which does not give the full picture of which group of participants' views have been reported. Being as specific as possible will make it easier for the reader to understand the findings.

2. While technical/implementing partners were part of the focus groups, no quotes from these participants have been presented in the manuscript. It is suggested to include some quotes from this group as well.

SUPPLEMENTARY MATERIAL:

1. It is recommended to add an appendix with codes, sub-themes/categories, themes, corresponding quotes.

CHECKLIST: Planning for and Assessing Rigor in Rapid Qualitative Analysis (PARRQA): consensus-based framework for designing, conducting and reporting

1. This might be a helpful resource to help with revisions: https://pmc.ncbi.nlm.nih.gov/articles/PMC11468362/

**********

what does this mean?). If published, this will include your full peer review and any attached files.

**Do you want your identity to be public for this peer review?**  If you choose “no”, your identity will remain anonymous but your review may still be made public.

For information about this choice, including consent withdrawal, please see our Privacy Policy

Reviewer #1: No

Reviewer #2: **Yes:**  Nikita Surani

**********

[NOTE: If reviewer comments were submitted as an attachment file, they will be attached to this email and accessible via the submission site. Please log into your account, locate the manuscript record, and check for the action link "View Attachments". If this link does not appear, there are no attachment files.]

Attachment Submitted filename: PLOS GPH_April 2026_Reviewer Comments_PGPH-D-26-01044.pdf

# Author comment: Author response to Decision Letter 1

**DOI:** 10.1371/journal.pgph.0006244.r002

29 Jun 2026

Attachment Submitted filename: Response to reviewers.docx

# Peer review: Decision Letter 1

**DOI:** 10.1371/journal.pgph.0006244.r003

**Authors:** Md. Alamgir Hossain

23 Sep 2026

Smart Paper Technology for maternal and newborn health data in The Gambia: a qualitative study of early implementation

PGPH-D-26-01044R1

Dear Oluwatosin Oizamsi Olumide Nkereuwem,

We are pleased to inform you that your manuscript 'Smart Paper Technology for maternal and newborn health data in The Gambia: a qualitative study of early implementation' has been provisionally accepted for publication in PLOS Global Public Health.

Before your manuscript can be formally accepted you will need to complete some formatting changes, which you will receive in a follow up email. A member of our team will be in touch with a set of requests.

Please note that your manuscript will not be scheduled for publication until you have made the required changes, so a swift response is appreciated.

IMPORTANT: The editorial review process is now complete. PLOS will only permit corrections to spelling, formatting or significant scientific errors from this point onwards. Requests for major changes, or any which affect the scientific understanding of your work, will cause delays to the publication date of your manuscript.

If your institution or institutions have a press office, please notify them about your upcoming paper to help maximize its impact. If they'll be preparing press materials, please inform our press team as soon as possible -- no later than 48 hours after receiving the formal acceptance. Your manuscript will remain under strict press embargo until 2 pm Eastern Time on the date of publication. For more information, please contact globalpubhealth@plos.org.

Thank you again for supporting Open Access publishing; we are looking forward to publishing your work in PLOS Global Public Health.

Best regards,

Md. Alamgir Hossain, M.S.S., M.P.H.

Academic Editor

PLOS Global Public Health

***********************************************************

Reviewer Comments (if any, and for reference):

The comment of Reviewer 3 is optional and can be ignored "The authors should consider reducing the length of the manuscript." No revisions are required.

Reviewer’s Responses to Questions

**Comments to the Author**

Reviewer #3: All comments have been addressed

Reviewer #4: (No Response)

**********

publication criteria?

Reviewer #3: Yes

Reviewer #4: Yes

**********

3. Has the statistical analysis been performed appropriately and rigorously?

Reviewer #3: Yes

Reviewer #4: Yes

**********

4. Have the authors made all data underlying the findings in their manuscript fully available (please refer to the Data Availability Statement at the start of the manuscript PDF file)?

The PLOS Data policy

Reviewer #3: Yes

Reviewer #4: Yes

**********

5. Is the manuscript presented in an intelligible fashion and written in standard English?

Reviewer #3: Yes

Reviewer #4: Yes

**********

Reviewer #3: The authors should consider reducing the length of the manuscript.

Reviewer #4: the authors have ensured that the results reflect the methodology and have clearly articulated the strengths and limitations of the study. Notably, the conclusion is also aligned.

**********

what does this mean?). If published, this will include your full peer review and any attached files.

**Do you want your identity to be public for this peer review?**  If you choose “no”, your identity will remain anonymous but your review may still be made public.

For information about this choice, including consent withdrawal, please see our Privacy Policy

Reviewer #3: No

Reviewer #4: No

**********
